# Reducing ER stress with chaperone therapy reverses sleep fragmentation and cognitive decline in aged mice

**DOI:** 10.1111/acel.13598

**Published:** 2022-04-30

**Authors:** Jennifer M. Hafycz, Ewa Strus, Nirinjini Naidoo

**Affiliations:** ^1^ 6572 Chronobiology and Sleep Institute and Department of Medicine Perelman School of Medicine University of Pennsylvania Philadelphia Pennsylvania USA

**Keywords:** aging, anti‐aging, behavior, molecular biology of aging, mouse models, neuroscience

## Abstract

As the aging population grows, the need to understand age‐related changes in health is vital. Two prominent behavioral changes that occur with age are disrupted sleep and impaired cognition. Sleep disruptions lead to perturbations in proteostasis and endoplasmic reticulum (ER) stress in mice. Further, consolidated sleep and protein synthesis are necessary for memory formation. With age, the molecular mechanisms that relieve cellular stress and ensure proper protein folding become less efficient. It is unclear if a causal relationship links proteostasis, sleep quality, and cognition in aging. Here, we used a mouse model of aging to determine if supplementing chaperone levels reduces ER stress and improves sleep quality and memory. We administered the chemical chaperone 4‐phenyl butyrate (PBA) to aged and young mice, and monitored sleep and cognitive behavior. We found that chaperone treatment consolidates sleep and wake, and improves learning in aged mice. These data correlate with reduced ER stress in the cortex and hippocampus of aged mice. Chaperone treatment increased p‐CREB, which is involved in memory formation and synaptic plasticity, in hippocampi of chaperone‐treated aged mice. Hippocampal overexpression of the endogenous chaperone, binding immunoglobulin protein (BiP), improved cognition, reduced ER stress, and increased p‐CREB in aged mice, suggesting that supplementing BiP levels are sufficient to restore some cognitive function. Together, these results indicate that restoring proteostasis improves sleep and cognition in a wild‐type mouse model of aging. The implications of these results could have an impact on the development of therapies to improve health span across the aging population.

## Graphical abstract

With age, there is a decline in sleep quality and cognitive ability that is associated with increased ER stress, which results in PERK activation and attenuated protein translation. Systemic administration of small molecule chaperone, PBA, improves sleep and cognition in aged mice by reducing PERK activation and GADD34, that is correlated with increased levels of p‐CREB. Local hippocampal overexpression of endogenous chaperone, BiP, is sufficient to reduce PERK activation, restore CREB activity and improve cognition.

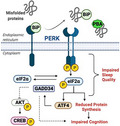

## 1 INTRODUCTION

The aging population is growing due to increased life expectancy, better health care, and improved socioeconomic development (Kanasi et al., 2016; United Nations, 2017). However, age is a risk factor for many health impairments and disorders (National Institute on Aging, 2007; United Nations, 2017). In particular, it has been well‐documented that sleep quality and cognitive ability both decline with age (Gulia & Kumar, 2018; Kumar, 2011; Soltani et al., 2019; Wimmer et al., 2012). Age‐related alterations in sleep include increased fragmentation and reduction in slow wave sleep in humans (Helfrich et al., 2018), and in mice (Franken et al., 2001; Naidoo et al., 2008; Wimmer et al., 2013), as well as an inability to sustain sleep and wake states in mice (Naidoo et al., 2008; Wimmer et al., 2013). These changes are conserved across various species (Brown et al., 2014; Koh et al., 2006; Mander et al., 2017; Mendelson & Bergmann, 1999; Naidoo et al., 2008, 2011; Pandi‐Perumal et al., 2002; Vienne et al., 2016; Welsh et al., 1986; Wolkove et al., 2007). Further, cognitive deficits associated with aging have been correlated with poor sleep and include disruptions in working memory and long‐term memory (Helfrich et al., 2018; Nebes et al., 2009; Schmutte et al., 2007). Given these age‐related changes in behavior, determining what alterations occur at the cellular level is vital in understanding the aging process as well as providing information that could lead to the development of therapies to improve health span.

With age, mechanisms that maintain cellular proteostasis are also less effective and less efficient. Specifically, there is an increase in endoplasmic reticulum (ER) stress with age (Brown et al., 2014; Naidoo et al., 2008, 2011). ER stress occurs when newly synthesized proteins misfold and aggregate in the ER lumen (Berridge, 2002; Lee et al., 2002; Ron & Walter, 2007; Szegezdi et al., 2006). When healthy organisms are in a state of ER stress, for example with sleep deprivation (Cirelli & Tononi, 2000; Naidoo et al., 2005, 2007; Tononi & Cirelli, 2003), the unfolded protein response (UPR) is acutely active and works to restore proteostasis by alleviating the protein folding load on the cell (Hetz et al., 2020; Kaufman, 2002; Koga et al., 2011), through the activation of the 3 UPR signaling sensors, PERK, IRE1, and ATF6. However, with age the UPR is less efficient/impaired and chronically activated, which leads to inflammatory signaling and cell death (Brown et al., 2014; Brown & Naidoo, 2012; Hetz & Mollereau, 2014; Koga et al., 2011; Naidoo et al., 2008). Under aging conditions, this UPR deficiency is coupled with a reduction in levels of the endogenous ER protein chaperone binding immunoglobulin protein (BiP), which is crucial to prevent protein aggregation (Brown et al., 2014; Hetz et al., 2020; Naidoo et al., 2008, 2011, 2018; Paz Gavilan et al., 2006).

PERK activation, which attenuates protein translation, has been linked to both sleep and cognition (Ly et al., 2020; Sharma et al., 2018). We have shown that PERK inhibition reduces sleep while PERK activation promotes sleep (Ly et al., 2020). PERK autophosphorylation leads to its activation. P‐PERK then phosphorylates eukaryotic initiation factor 2alpha (eIF2α) preventing the formation of the ternary ribosomal complex, which halts global protein translation, allowing only a small subset of transcripts required to maintain proteostasis to be translated (Ron & Walter, 2007). The translation block is terminated by GADD34 activation downstream of PERK and Activating transcription factor 4 (ATF4). GADD34 dephosphorylates p‐eIF2a and functions as a negative feedback loop for PERK activation. Chronic PERK activation leads to apoptosis and inhibited protein translation, which has many consequences, including memory deficits (Hughes & Mallucci, 2019; Hussain & Ramaiah, 2007; Sen et al., 2017; Sharma et al., 2018).

In an earlier study, we demonstrated that supplementing endogenous chaperone levels with the chemical chaperone, 4‐phenyl butyrate (PBA), in aged wild‐type drosophila reverses age‐related sleep fragmentation, as well as reduces ER stress and UPR activity (Brown et al., 2014). In this study, we examine whether a similar treatment in aged mice would lead to improved proteostasis and sleep quality. Further, we asked whether PBA treatment would ameliorate age‐related cognitive deficits.

Thus, we treated young and aged mice with PBA or saline over an extended period and subsequently monitored sleep and cognitive behavior. We report that PBA treatment ameliorates age‐related behavioral phenotypes. PBA treatment also led to a diminution of ER stress markers and an increase in CREB activation in the brains of aged mice. Lastly, we overexpressed BiP in the hippocampus of aged mice and observed improved cognition, reduced ER stress, and increased CREB activation in the hippocampi of aged mice. The results garnered from these experiments could have an impact on the development of therapies for sleep quality improvement and reduction of cognitive deficits in aged individuals, which could together improve the health span of the growing aged population.

## 2 RESULTS

### 2.1 PBA treatment consolidates sleep and wake states in aged mice

**FIGURE 1:**
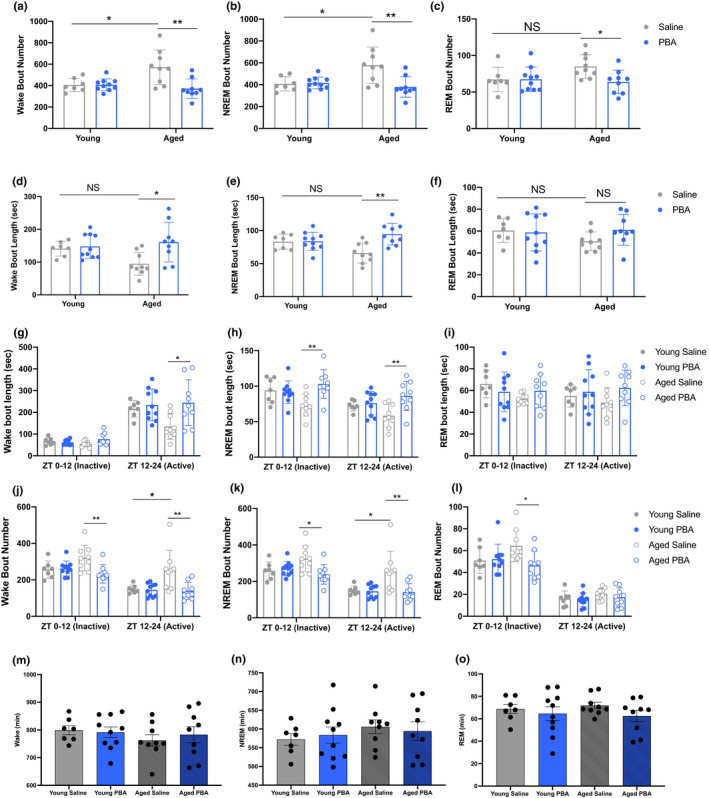
PBA treatment consolidates sleep and wake in aged mice. (a) Wake bout number (b) NREM bout number (c) REM bout number; (a‐c average bout number over 24 h ± SEM); (d) Wake bout length (e) NREM bout length (f) REM bout length (d‐f average bout length (seconds) over 24 h) (g) Wake bout length (h) NREM bout length (i) REM bout length (g‐i average sleep and wake bout length (seconds) between lights on (ZT0‐12) and lights off (ZT12‐24) periods); (j) Wake bout number (k) NREM bout number (l) REM bout number (j‐l average bout numbers between lights on (ZT0‐12) and lights off (ZT12‐24) periods); (m) Total Wake, (n) Total NREM, (o) Total REM (m‐o total sleep and wake (minutes) during 24 h period) (*p < 0.05, **p < 0.01, ***p < 0.001, two‐way ANOVA with Tukey post hoc correction for multiple comparisons; *n* = 7 young saline, *n* = 10 young PBA, *n* = 9 aged saline, *n* = 9 aged PBA)

We have previously demonstrated that PBA administration in aged drosophila reduced ER stress and consolidated sleep in these flies (Brown et al., 2014). Whether this amelioration of poor sleep quality by chaperone administration could be replicated in a mammalian model was not known. We therefore examined the effect of 10 weeks of PBA treatment on sleep‐wake behavior in young (2mo) and aged (18mo) mice using electroencephalogram (EEG) recordings. Control mice were administered saline over the 10‐week period and subjected to the same behavioral assays. We examined sleep‐wake characteristics across 24hs as well as during 12h light: 12h dark periods. First, we were able to recapitulate previously published data (Naidoo et al., 2008, 2011; Wimmer et al., 2013) that indicate aged mice have increased sleep fragmentation during the inactive and active period, as well as wake fragmentation during the active period relative to young mice (Figure 1, Tables S1–S3).

In the 24 h analyses, we found that aged saline‐treated mice had a greater number of wake bouts compared to young saline‐treated mice (*p *< 0.05; Figure 1a, Table S2), indicating fragmentation of wake. PBA treatment reduced the number of wake bouts and increased the duration of these bouts in aged mice (*p *< 0.01, *p *< 0.05, respectively; Figure 1a, d, Table S2), thus indicating consolidation of wakefulness. Sleep, like wake, was fragmented in aged saline‐treated mice, with more NREM bouts (*p *< 0.05, Figure 1b) relative to young saline‐treated mice across the 24hr day (Table S2). PBA treatment reduced the number of NREM bouts and lengthened the duration of these bouts (*p *< 0.01 and *p *< 0.01, respectively; Figure 1b, e; Table S2). Lastly, in the 24hr period, PBA‐treated aged mice had a reduced number of REM bouts compared to aged saline mice (*p *< 0.05, Figure 1c, Table S2). Thus, PBA treatment consolidated both NREM and REM sleep in aged mice.

To obtain a more detailed description of changes in sleep and wake behavior, we also analyzed the data during the lights on (sleep or inactive) and lights off (active) periods in 12 h bins. We found that aged mice had fragmented wake compared to young mice and that PBA treatment consolidated wake in aged mice, particularly at night, which is the normal waking period for these animals. Specifically, aged mice displayed more wake bouts during the lights off period compared to young mice (*p *< 0.05; Table S3, Figure 1j). PBA treatment in aged mice reduced wake bout numbers during the lights on and lights off periods compared to aged saline mice (*p *< 0.01, *p *< 0.01; Table S3, Figure 1j) and increased wake bout length during the night (active period) compared to aged saline mice (*p *< 0.05, Table S3, Figure 1g).

As described for wake, sleep in aged mice is fragmented, with more NREM bouts in the lights off period (*p *< 0.05, Table S3, Figure 1k). PBA‐treated aged mice displayed an increase in NREM bout duration at both lights on and off periods compared to aged saline mice (*p *< 0.01 and *p *< 0.01; Table S3, Figure 1h) as well as a reduction in the number of NREM bouts during both lights on and lights off compared to aged saline‐treated mice (*p *< 0.05 and *p *< 0.01, respectively; Table S3, Figure 1k). Lastly, with PBA treatment, aged mice had fewer REM bouts during lights on compared to aged saline mice (*p *< 0.05, Table S3, Figure 1l). Together, these data indicate that PBA treatment consolidated both REM and NREM sleep.

Aging has been shown to reduce the homeostatic response to sleep loss (Hasan et al., 2012), as well as reduce peak theta power and slow wave activity (Wimmer et al., 2013). We examined the recovery sleep response to 6hrs of sleep deprivation and found no significant effect of age or treatment on total NREM sleep following sleep deprivation (Figure S1A). Spectrogram analyses of the EEG recordings of NREM, REM, and wake indicated that there were no effects of age or treatment (Figure S1A‐E). We found no differences in delta power (0–4 Hz) or peak theta (6–8 Hz) (data not shown) between aged saline‐treated mice and young saline‐treated mice, nor were there any changes with PBA treatment (Tables S4 and S5). Further, no changes were observed in the intensity of delta rebound or in the swiftness of delta discharge following sleep deprivation, indicating that chaperone treatment did not alter the homeostatic response to sleep loss (Figure S1F).

### 2.2 PBA reduces ER stress in the cerebral cortex of aged mice

It is known that PBA treatment reduces UPR activity in aged drosophila brains (Brown et al., 2014) and protects against ER stress in mouse brains (Li et al., 2019) and pancreas tissue (Guo et al., 2017; Ozcan et al., 2006). Using immunofluorescence, we examined the effect of PBA treatment on ER stress in the cerebral cortex in young and aged mice as the cortex is known to regulate sleep and wake (Krone et al., 2021; Muzur et al., 2002; Naidoo et al., 2011) Thus, molecular changes in the cortex could be reflected in behavior. We specifically focused on the PERK pathway, given the role for PERK in sleep regulation. We have previously shown in drosophila that PERK activation under unstressed conditions increases sleep while PERK inhibition reduces sleep (Ly et al., 2020). We have also demonstrated that increased ER stress and chronic PERK activation in aging is associated with poor sleep and wake quality (Brown et al., 2014; Naidoo et al., 2008, 2011).

**FIGURE 2:**
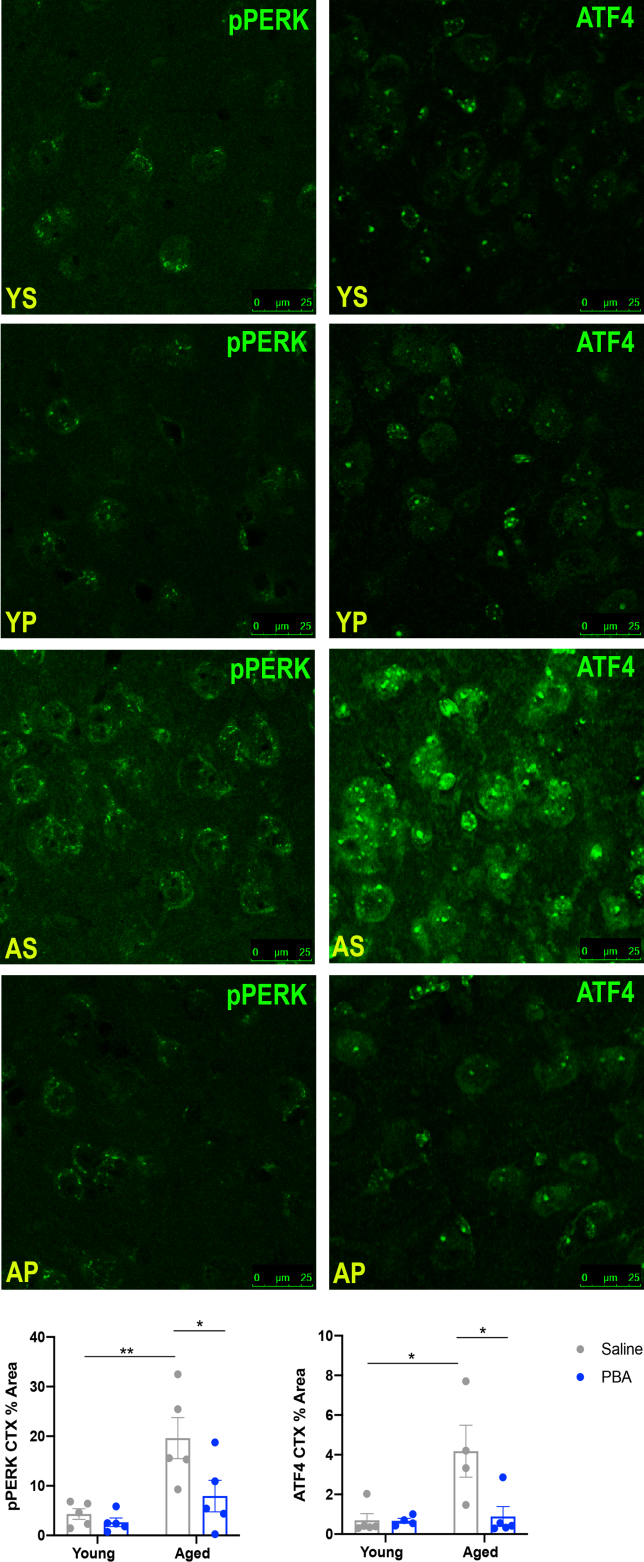
PBA treatment reduces UPR activity in the cortex of aged mice. Confocal images of cortex across groups. Left: p‐PERK; Right: ATF4. Below: Mean ± SE percent area of p‐PERK and ATF4 within cell bodies in cortex (*n* = 4–5 animals per group; two‐way ANOVA with Tukey post hoc correction for multiple comparisons, *p < 0.05, **p < 0.01; Abbr: Young Saline = YS; Young PBA = YB; Aged Saline = AS; Aged PBA = AP)

We found that saline‐treated aged mice displayed more ER stress and UPR activity in the cerebral cortex when compared to young mice (Figure 2). Specifically, we observed increased phosphorylation of PERK and of ATF4, a downstream target of activated PERK (*p *< 0.05, Figure 2). PBA had no effect on PERK activation in young mice, which remained low (Figure 2). However, PBA treatment reduced PERK activation and ATF4 staining in aged mice (*p *< 0.05, Figure 2), consistent with previous findings and indicating that ER stress is ameliorated with chaperone administration.

As PBA treatment consolidated sleep and wake in aged mice, we wanted to examine any effect of PBA treatment in orexin neurons, which are responsible for maintenance of state (Alexandre et al., 2013; Mochizuki et al., 2004). We counted orexin neurons and calculated the percentage that were p‐PERK‐positive (Figure S2) and found that there were no significant differences in any groups (Figure S2).

### 2.3 PBA administration improves cognitive performance in aged mice

**FIGURE 3:**
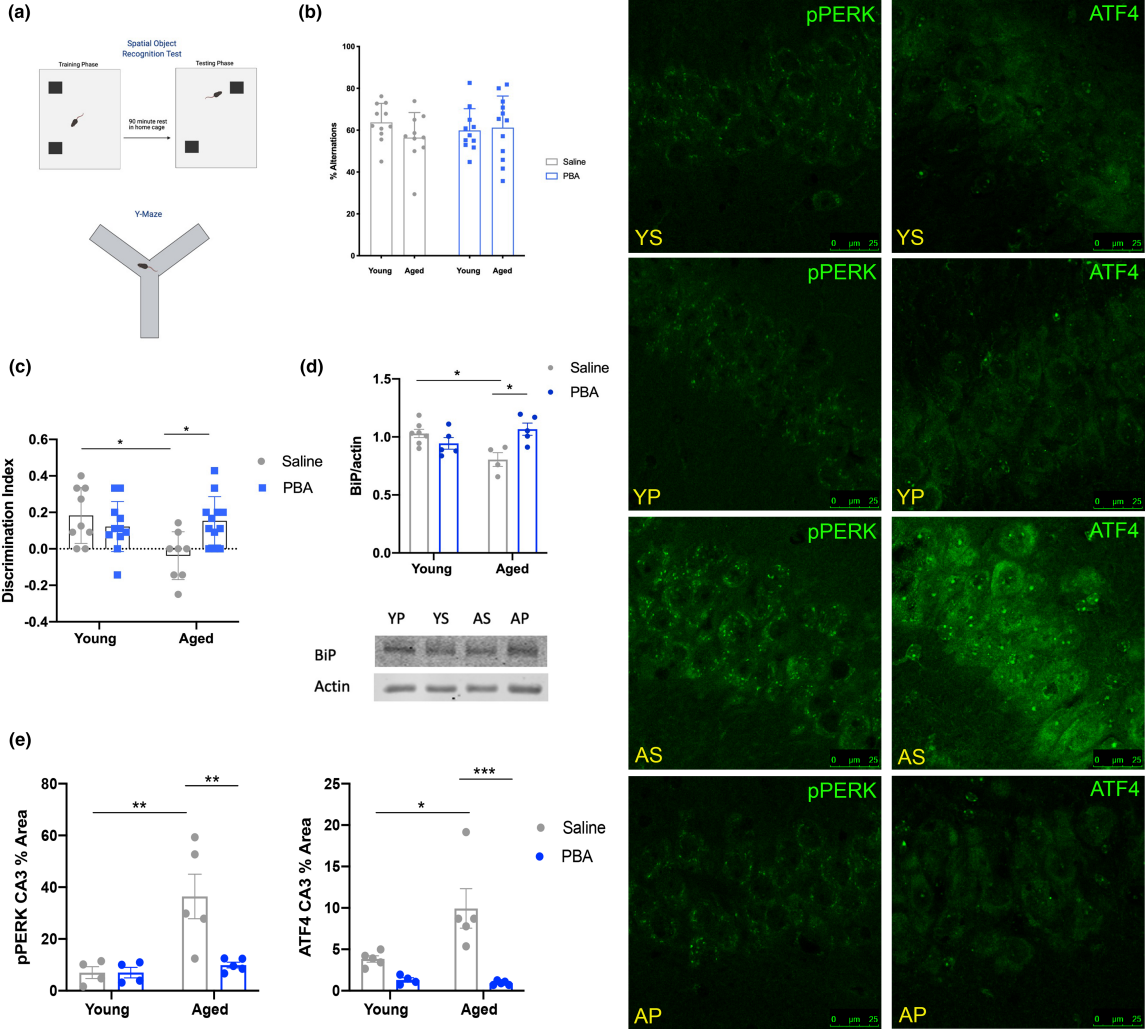
Chaperone treatment improves performance in the SOR test and reduces hippocampal p‐PERK in aged mice. (a) Schematic of SOR and Y‐maze tests; (b) Percent alternations from Y‐maze test; (c) Discrimination index of SOR test; (d) Western blot analysis of BiP levels in all groups, representative blot image below; (e) Mean ± SE percent area of p‐PERK (left) and ATF4 (right) in the CA3 region of the hippocampus. (*p < 0.05, **p < 0.01, ***p < 0.001, two‐way ANOVA with Tukey post hoc correction for multiple comparisons; n=8–10 SOR test) Left: confocal images (*n* = 4–5/group; Abbr: Young Saline = YS; Young PBA = YP; Aged Saline = AS; Aged PBA = AP)

In addition to worsening sleep quality, cognitive ability also declines with age (Nebes et al., 2009; Ohayon & Vecchierini, 2002; Wimmer et al., 2012). To determine if chaperone treatment impacts cognition in this model of aging, young and aged mice were subjected to two hippocampal‐dependent cognitive tasks, the spatial object recognition test (SOR) and the Y‐maze test (Figure 3a). To quantify learning in the SOR test, a discrimination index was calculated, to determine the amount of time spent with the moved object compared to the unmoved object (Chuluun et al., 2020; Haettig et al., 2011).

Young mice exhibited a strong preference for the moved object (discrimination index) in the SOR test, regardless of treatment (Figure 3c). Aged mice that received only saline injections could not discriminate between the moved and unmoved object compared to young saline‐treated mice (*p *< 0.05), thus demonstrating impaired cognition (Figure 3c). With PBA treatment, aged mice exhibited an improved ability to discriminate between the two objects, preferring the moved object, compared to aged saline mice (*p *< 0.05). PBA‐treated aged mice performed indistinguishably from young mice, indicating that PBA treatment rescued the cognitive impairment (Figure 3c).

Concurrently with the SOR test, young and aged mice were subjected to the working memory Y‐maze test. A good working memory is evident via repeated complete alternations, entering into each of the three arms of the apparatus consecutively (Figure 3a). All groups performed variably in this test, and there were no clear differences in performance, regardless of age, or treatment (Figure 3b).

Interestingly, 66.67% of the PBA‐treated aged mice that displayed improved performance in the SOR test (i.e., passed the test) also had improved sleep consolidation (greater average NREM bout duration and lower NREM bout numbers) compared to the aged saline‐treated mice. This suggests that with PBA treatment, there is a positive correlation between consolidated sleep and improved cognition.

### 2.4 PBA treatment reduces UPR activity specifically in the hippocampus of aged mice

To understand how learning was improved by chaperone treatment, we used immunofluorescence to examine the effect of treatment on the expression of ER stress markers in the hippocampus. We first probed for the endogenous chaperone protein BiP via Western blot assay and found that aged saline‐treated mice had less BiP than young mice, recapitulating results from previous studies (*p *< 0.05, Figure 3d; Naidoo et al., 2008, 2018). PBA treatment in aged mice rescued BiP levels (*p *< 0.05, Figure 3d). Further, we found that aged saline‐treated mice displayed more ER stress and UPR activity in the CA3 region of the hippocampus, with increased p‐PERK and ATF4 staining relative to young mice (*p *< 0.01 and *p *< 0.05, respectively, Figure 3e). With PBA treatment, there is a significant reduction in the expression of p‐PERK and its downstream target ATF4 in CA3 in aged mice (*p *< 0.01 and *p *< 0.001, respectively, Figure 3e).

### 2.5 PBA treatment increases expression of p‐CREB

Given the effect of PBA treatment on cognitive performance in aged mice in the SOR test, we determined whether PBA treatment was correlated with changes in the expression of markers of synaptic plasticity. Phosphorylated CREB is a well‐known marker of synaptic plasticity and its activation is associated with memory formation, while its inhibition or downregulation is associated with disrupted memory (Bourtchuladze et al., 1994; Xiong et al., 2013; Yu et al., 2017). CREB is a transcription factor that drives the expression of key proteins involved in memory, like BDNF, which plays a role in synaptic function and memory processes (Gonzalez et al., 2019; Panja & Bramham, 2014). While any changes in p‐CREB could reflect other molecular alterations, given the role of p‐CREB in memory, we sought to determine if PBA treatment and improved SOR performance correlated with increased p‐CREB staining.

**FIGURE 4:**
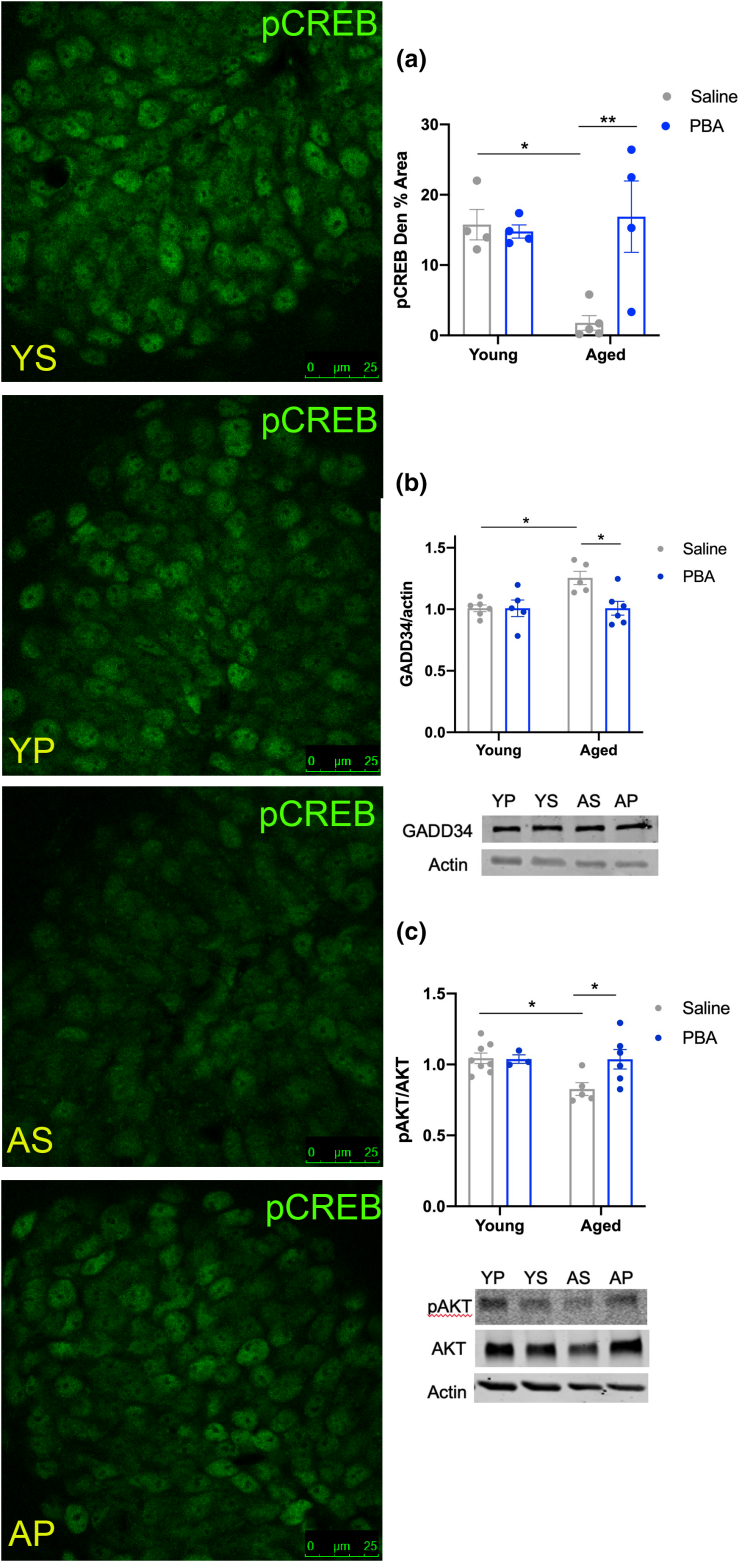
PBA treatment increases p‐CREB in the dentate gyrus of aged mice and increases p‐AKT in the hippocampus of aged mice. Confocal images of hippocampus with p‐CREB immunostaining across all four groups; (a) Mean ± SE percent area of p‐CREB within cell bodies in the dentate gyrus of the hippocampus (*n* = 4–5 animals per group); (b) Western blot analysis of GADD34, representative images below (YS *n* = 6, AS *n* = 9, AP *n* = 6); (c) Ratio of p‐AKT/AKT using actin as a loading control, representative blots below (YS *n* = 8, AS *n* = 7, AP *n* = 8). (Data presented as mean ± SE; all quantifications analyzed via two‐way ANOVA with Tukey post hoc correction for multiple comparisons, *p < 0.05, **p < 0.01; Abbr: Young Saline = YS; Aged Saline = AS; Aged PBA = AP)

Immunofluorescence staining of hippocampal sections for CREB and p‐CREB indicated that aged saline‐treated mice had reduced p‐CREB levels compared to young mice, particularly evident in the dentate gyrus (*p *< 0.05, Figure 4a). PBA treatment had no apparent effect on p‐CREB in young mice, though aged mice that were treated with PBA displayed increased hippocampal p‐CREB relative to saline‐treated aged mice (*p *< 0.01, Figure 4a).

We next determined the mechanism linking ER stress and UPR activity to the changes observed in p‐CREB levels. It has been shown that GADD34, a phosphatase downstream of PERK that dephosphorylates eIF2α (Tsaytler & Bertolotti, 2013), also inhibits AKT (protein kinase B), a CREB kinase (Du & Montminy, 1998; Farook et al., 2013; Sen, 2019).

We therefore probed levels of GADD34, p‐AKT, and AKT in the hippocampi of young and aged mice via Western blot assays to determine whether p‐AKT was altered with drug treatment. Western blot analyses revealed that there was an increase in GADD34 in aged saline mice compared to young saline mice (*p *< 0.05, Figure 4b). Further analyses revealed a reduction in GADD34 in the hippocampi of aged PBA‐treated mice compared to aged saline‐treated mice (*p *< 0.05, Figure 4b). We also observed that aged saline‐treated mice had less p‐AKT than young mice (*p *< 0.05). PBA treatment in aged mice increased p‐AKT expression compared to aged saline‐treated mice (*p *< 0.05, Figure 4cp), suggesting that PBA treatment led to increased AKT activation in aged mice.

### 2.6 Binding Immunoglobulin Protein (BiP) overexpression in the hippocampus improves proteostasis, cognition, and CREB activity in the hippocampus of aged mice

Having observed the benefit of systemic administration of chaperone treatment on sleep and learning, we wanted to determine whether specifically increasing chaperone levels in the hippocampus would be sufficient to improve learning in aged mice. We therefore overexpressed BiP in the hippocampus using an AAV‐CaMKII‐BiP viral vector. Control mice received an AAV‐CaMKII‐mCherry vector microinjection.

**FIGURE 5:**
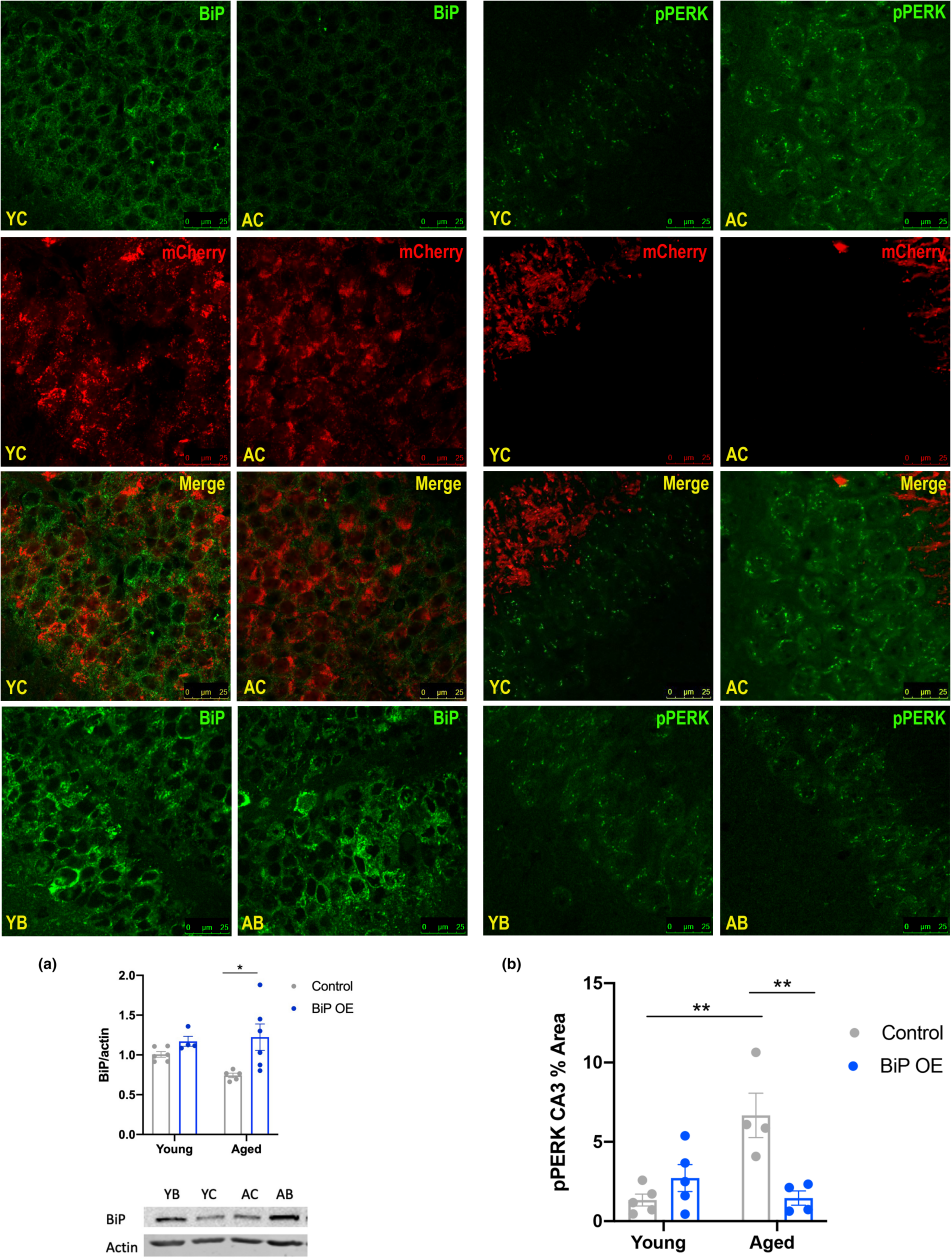
Hippocampal BiP overexpression reduces p‐PERK staining in aged mice. Confocal images of hippocampal BiP staining (left, green) and p‐PERK (right, green) in young and aged mice. Merged images are shown for mCherry‐tagged (red) control virus‐injected mice. (a) Western blot analysis of BiP; images below (*n* = 5 for all groups). (b) Mean ± SE percent area of p‐PERK in CA3 region of the hippocampus (*n* = 4–5 animals per group; all quantifications were analyzed via two‐way ANOVA with Tukey post hoc correction for multiple comparisons, *p < 0.05, **p < 0.01; Abbr: Young Control = YC; Young BiP = YB; Aged Control = AC; Aged BiP = AP)

BiP overexpression was confirmed by immunostaining and Western blot (Figure 5 and Figure S3). BiP expression was markedly increased in the dentate gyrus (Figure 5) and CA3 region (Figure S3). Control vector injection was also confirmed with prominent mCherry fluorescence visible in the dentate gyrus (Figure 5) and CA3 of the hippocampus (Figure S3). BiP immunostaining was quantified with Western blot assays in bulk hippocampal tissue where we observed an increase in BiP expression in aged AAV‐BiP mice relative to AAV‐control aged mice (*p *< 0.05, Figure 5a).

Hippocampal tissue in these mice was examined via immunofluorescence to probe markers of ER stress, with a focus on the PERK pathway. Young mice regardless of virus appeared to have little PERK activation in the CA3 region with minimal visible p‐PERK staining (Figure 5). Aged AAV‐control mice displayed more activated PERK (p‐PERK) relative to young AAV‐control mice (*p *< 0.01, Figure 5b). Critically, BiP overexpression in aged mice had reduced p‐PERK staining relative to AAV‐control aged mice, most clearly in the CA3 region of the hippocampus (*p *< 0.01, Figure 5b).

**FIGURE 6:**
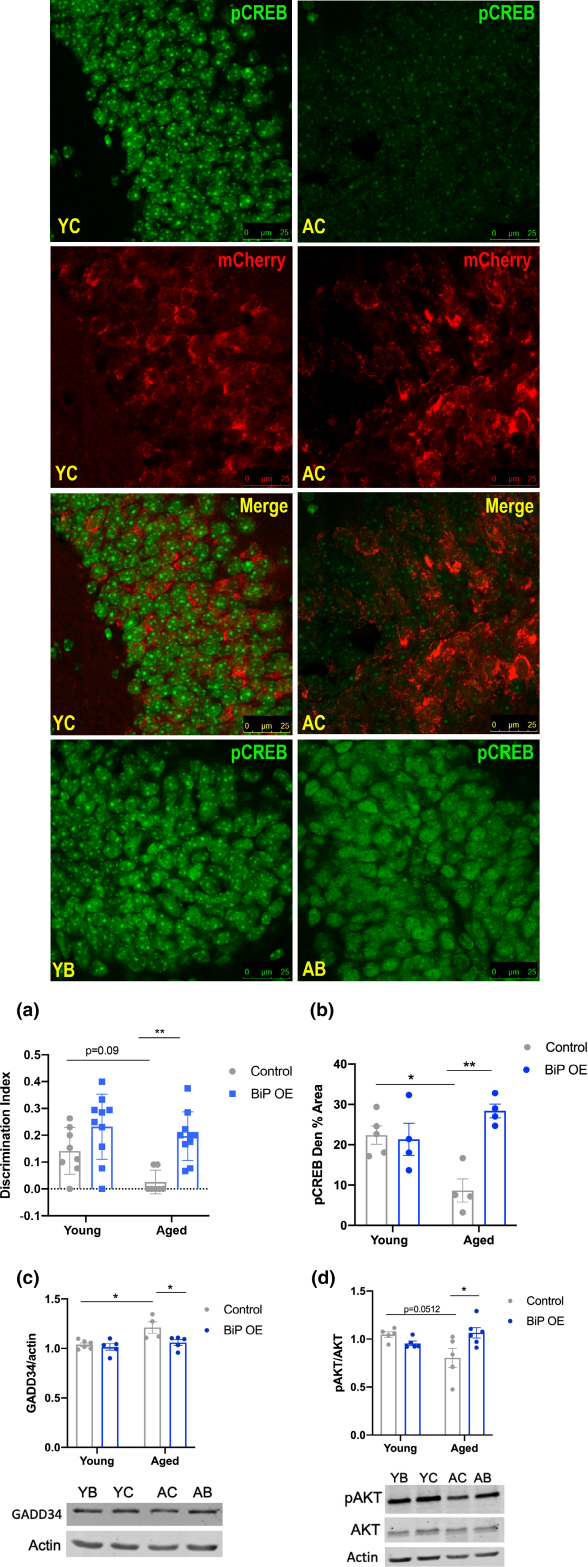
Hippocampal BiP overexpression increases p‐CREB and p‐AKT in aged mice. Confocal images of p‐CREB (green) staining across all four groups. Merged images are shown for mCherry‐tagged (red) control virus‐injected mice (*n* = 5–8 for all groups). (a) Discrimination index from SOR test (*n* = 8–10 for all groups); (b) Mean ±SE percent area of p‐CREB in the dentate gyrus of the hippocampus (*n* = 4–5 animals per group); (c) Western blot quantification of GADD34, images below (*n* = 5–6 animals per group); (d) Ratio of p‐AKT/AKT using actin as a loading control, images below (*n* = 4–6 animals per group); (Data presented as mean ± SE; all analyses were done via two‐way ANOVA with Tukey post hoc correction for multiple comparisons *p < 0.05, **p < 0.01, ***p < 0.001, Abbr: YC = young control, YB = young BiP, AC = aged control, AB =aged BiP)

Following BiP viral overexpression, we subjected all mice to the SOR and Y‐maze cognitive tasks. Young mice displayed a preference for the moved object in the SOR test regardless of viral injection (Figure 6a). Aged mice that received AAV‐BiP were able to discriminate between the moved and unmoved objects, preferring the moved object relative to aged AAV‐control injected mice (*p *< 0.01, Figure 6a), indicative of improved cognition. Aged mice that received AAV‐BiP injections performed similarly to young mice. However, as with PBA treatment, young and aged mice performed variably in the Y‐Maze test, regardless of age or which virus was injected (data not shown).

We examined p‐CREB levels to determine if BiP overexpression led to an increase in p‐CREB in aged mice, as with PBA treatment. Aged AAV‐control mice had less p‐CREB compared to young AAV‐control mice which was clearly evident in the dentate gyrus (*p *< 0.05, Figure 6b). With BiP overexpression, aged mice displayed an increase in CREB activation relative to aged AAV‐control mice (*p *< 0.01, Figure 6b), indicating that BiP overexpression in the hippocampus is sufficient to restore CREB activation in aged mice. We also probed GADD34 and AKT activation to determine if CREB levels were altered in AAV‐BiP aged mice by a mechanism similar to that observed with PBA treatment. As with the PBA study, we found that aged AAV‐control mice had more GADD34 and less p‐AKT in the hippocampus compared to young mice (*p *< 0.05 and *p *< 0.0512, respectively, Figure 6c‐d). AAV‐BiP‐injected mice displayed a significant reduction in GADD34 and a significant increase in p‐AKT compared to that in aged AAV‐control mice (*p *< 0.05, Figure 6c‐d).

Altogether, these data suggest that increasing endogenous chaperone levels locally in the hippocampus in aged mice is sufficient to partially restore proteostasis in these brain regions, as observed via a reduction in PERK activation. BiP overexpression is further correlated with improved cognition in aged mice and increased CREB and AKT activation.

## 3 DISCUSSION

In this study, we postulated that restoring proteostasis via chaperone treatment would consolidate sleep and improve cognition in aged mice. We have shown that treatment with the chemical chaperone, PBA, consolidates sleep and wake in aged mice, recapitulating the results from drosophila studies (Brown et al., 2014). With age, sleep and wake are fragmented (Naidoo et al., 2008, 2011; Wimmer et al., 2013), which we observed again here in the control aged saline‐treated mice. PBA treatment consolidated behavioral state in aged mice, which was correlated with reduced UPR activity, specifically PERK activation, in the cortex. Importantly, we note that PBA is also a histone deacetylase (HDAC) inhibitor (Ricobaraza et al., 2009). While we did not explore any effects of PBA as an HDAC inhibitor, we have conducted similar aging sleep characterization studies in drosophila using sodium butyrate, which acts only as an HDAC inhibitor and not a chaperone (Kim et al., 2007; Zhang et al., 2016), and found no changes in sleep with sodium butyrate treatment (data to be published). In addition to sleep and wake fragmentation, it has been well‐documented that age impairs rebound sleep and slow wave activity following sleep deprivation via gentle handling (Hasan et al., 2012; Mander et al., 2017; Wimmer et al., 2013). However, we were unable to recapitulate these effects under our experimental conditions. It is possible that object exploration sleep deprivation methods do not produce the same changes in rebound sleep and slow wave activity as gentle handling and mechanical sleep deprivation methods. It would be fascinating to compare the effects of different sleep deprivation methods on rebound sleep in future studies.

We have previously demonstrated that PERK signaling is involved in sleep regulation (Ly et al., 2020). In particular, that inhibiting PERK reduces sleep, while overexpressing PERK induces sleep (Ly et al., 2020). We have shown that PERK activation and the ensuing translational block through p‐eIF2α led to a decrease in translation of a wake promoting peptide in Drosophila (Ly et al., 2020). Phosphorylated eIF2α dependent inhibition of protein translation has been shown to promote sleep in mice (Methippara et al., 2009), likely though a similar mechanism. We think that PERK activation signals sleep in order to relieve the ER stress that ensues with wakefulness through phosphorylation of eIF2α and that the subsequent translational block is alleviated by GADD34, downstream of PERK, signaling wake. However, with age BiP, which negatively regulates the UPR, is reduced resulting in chronically activated UPR and PERK and in higher expression of GADD34 (Brown et al., 2014; Naidoo et al., 2008, 2011). The bidirectional effects of enhanced GADD34 and PERK on the phosphorylation status of eIF2α likely alters sleep state during aging. Further, chronic activation of the PERK pathway leads to apoptotic signaling, contributing to a maladaptive response and to poor cellular health in both aging and disease (Naidoo et al., 2005, 2008, 2011). Restoring proteostasis in the brains of aged mice via chaperone treatment contributes to the prevention of behavioral state fragmentation by reducing ER stress and PERK activation, allowing for the resumption of normal protein translation.

Sleep is thought to serve many different functions, one of which is the consolidation of memories (Aton et al., 2009; Diekelmann & Born, 2010; Seibt et al., 2012; Tudor et al., 2016). Critically, changes in sleep with age have been linked to poorer cognition; specifically that poor quality NREM sleep is linked to poor memory, as is sleep fragmentation and increased daytime sleepiness (Mander et al., 2017; Pandi‐Perumal et al., 2002; Welsh et al., 1986; Wolkove et al., 2007). Several other studies have shown a correlation between poor sleep quality with age and working memory, long‐term memory, verbal knowledge, and spatial reasoning (Nebes et al., 2009; Schmutte et al., 2007). Notably, in our experiments, the PBA‐treated aged mice that had improved sleep consolidation also performed well in the SOR test. However, a shortcoming of this study is that we only used male mice. In future planned experiments, females will be used as it is vital to determine if similar mechanisms affect sleep and cognition with age in female subjects. As discussed above, chronic activation of the UPR with age attenuates protein translation through PERK activation and p‐eIF2a (Brown et al., 2014; Naidoo et al., 2008, 2011) yet protein synthesis is necessary for memory formation and synaptic function (Hernandez & Abel, 2008; Seibt et al., 2012; Tudor et al., 2016). We have shown here that both global and local hippocampal chaperone treatment reducing PERK activity improved learning in aged mice. Reduction in PERK activation with chaperone treatment indicates that chaperone treatment de‐represses translation, thus allowing for learning in aged mice and is consistent with previous studies that show inhibiting or reducing PERK activation improves memory (Halliday & Mallucci, 2015; Halliday et al., 2015; Ma et al., 2013; Radford et al., 2015). PERK inhibition via GSK2606414 in a prion‐disease model reduced PERK activity and had neuroprotective effects (Moreno et al., 2012). BiP overexpression also reduced PERK activity in CA3 and CA1 but the most marked increase of BiP was observed in the dentate gyrus and CA3 regions of the hippocampus. It is possible that BiP overexpression in the dentate gyrus and CA3 was able to affect PERK activation in adjacent regions, as it has been established that modifying the UPR in one region can have effects on more distal regions (Taylor & Dillin, 2013), and it is well‐known that the dentate gyrus projects to CA3, which sends its projections to the CA1 (Lisman, 1999). We posit that the improved proteostasis with chaperone treatment, at both the local hippocampal level and global level, coupled with improved sleep quality, could both contribute to improved cognition in this model of aging. We acknowledge that a limitation of our study is that we did not extensively test cognitive behavior with a comprehensive set of tests; however, our data do support the idea that chaperone therapy improves learning in aged mice in the SOR test.

Coupled with reduced ER stress and PERK activity in the hippocampi of aged mice, we also observed that chaperone therapy increased p‐CREB levels in the hippocampi of aged mice. Phosphorylated CREB has been shown in various studies to be involved in memory processes (Blendy et al., 1996; Bourtchuladze et al., 1994; Graves et al., 2003; Yu et al., 2017; Zhou et al., 2013). CREB is a transcription factor that leads to the expression of several synaptic plasticity factors, such as BDNF, which is involved in memory formation (Bekinschtein et al., 2008; Gonzalez et al., 2019; Panja & Bramham, 2014; Sharma et al., 2021). CREB activation provides an interesting target molecule, particularly because the UPR, specifically PERK (Sen et al., 2017), has been shown to directly affect CREB activity. GADD34 could disrupt CREB activity by preventing its phosphorylation via p‐AKT (Sen, 2019) thus directly linking UPR activity to pathways involved in memory formation. Our results suggest that this might be the case, as we observed more p‐PERK and GADD34 in the hippocampus of control aged saline‐injected mice, accompanied by less p‐AKT and p‐CREB relative to young mice. With PBA, p‐PERK and GADD34 levels decrease, while p‐AKT and p‐CREB levels are increased. Similarly, BiP overexpression in the hippocampus also increased p‐AKT and p‐CREB. Thus, reducing ER stress via supplementing chaperone levels in the hippocampi of aged mice is sufficient to restore some cognitive function through increasing p‐AKT activity and p‐CREB levels. However, p‐CREB is an indirect measure of molecular changes and could reflect alterations in several signaling pathways. While GADD34 altering p‐AKT levels is a possible explanation for the changes observed in p‐CREB, there could be other factors at play. Because PERK activation ultimately leads to reduced global translation, it is highly likely that there are other kinases affected by this translation block that lead to diminished p‐CREB levels. PERK activation has been shown to directly affect parts of memory formation and consolidation pathways, and increased PERK activity has been associated with decreased synaptic strength and poor memory consolidation (Halliday & Mallucci, 2014; Havekes et al., 2012; Hetz & Mollereau, 2014; Sharma et al., 2018). It has also been shown that PERK activation and subsequent eIF2α phosphorylation impairs LTP (Costa‐Mattioli et al., 2007). It could be this collective dysfunction in protein quality control systems and sleep with age that together contribute to memory impairments via impacting pathways involved in memory processes.

Our results suggest that alleviating ER stress through chaperone therapy could improve health span in the growing aged population by consolidating sleep and by improving learning via restoring proteostasis in the brain.

## 4 METHODS

### 4.1 Mice

Animal experiments were conducted in accordance with the guidelines of the University of Pennsylvania Institutional Animal Care and Use Committee. Mice were maintained as previously described (Chellappa et al., 2019; Naidoo et al., 2008). Mice were housed at 23°C on a 12:12 h light/dark cycle and had ad libitum access to food and water. For PBA treatment studies, 47 male C57BL6 mice, 24 aged (18–22 months old) and 23 young (2–6 months old), were obtained from the National Institute of Aging. Survival and distribution of these mice are presented in Table S6. A separate set of 36 male C57BL6 mice were used for BiP viral overexpression experiments. All mice survived surgery and underwent cognitive testing.

### 4.2 Drug administration

Sodium 4‐phenyl butyrate (PBA) (Cayman Chemical, Ann Arbor, MI) was administered twice weekly via intraperitoneal (IP) injections at a dose of 40mg/kg and in the drinking water as a 0.8% PBA concentration in a 1% sucrose solution. This dose was determined by a comparison of doses used in similar studies and our own unpublished pilot data (Cao et al., 2016; Ricobaraza et al., 2011). Vehicle‐treated mice received IP sterile saline injections twice weekly and were given a 1% sucrose solution as drinking water. Treatment began when mice were 18 months old (aged) or 2–3 months old (young) and continued for 10–12 weeks. Following treatment, all mice were subject to electroencephalogram implant surgeries. After recovery, all mice underwent cognitive behavioral testing and sleep recordings. Mice were sacrificed via transcardial saline perfusions at ZT0 immediately following the recovery sleep period. Half brains were collected and either flash frozen or fixed in 4% paraformaldehyde.

### 4.3 Electroencephalogram (EEG) surgery and sleep recordings

Electroencephalogram surgeries were performed as previously described, with minor adjustments (Naidoo et al., 2018; Perron et al., 2015). Briefly, mice were anesthetized with isoflurane. Four EEG electrodes and two EMG electrodes were implanted and held in place with dental acrylic. After one week recovery from surgery, mice were individual housed and connected to EEG cables and acclimated for three days prior to the start of recordings. Recordings took place as previously described (Naidoo et al., 2018; Perron et al., 2015). Recordings started at ZT0 lights on (10:00 a.m.) and continued for two consecutive days. The first 24 h of recording served as baseline sleep recordings. During the second day of EEG recordings, from ZT0 to ZT6 (10:00 a.m. to 4:00 p.m.) the mice were sleep deprived via novel object presentation and gentle handling if necessary as previously described (Zhu et al., 2018). Mice were allowed 18 hours of recovery sleep from ZT6 to ZT0 (4:00 p.m. to 10:00 a.m.). Data were scored with SleepSign Analysis Software (Kissei Comtec Co., LTD), and spectral data were analyzed as previously described (Franken et al., 2001; Hasan et al., 2012; Lim et al., 2013; Naidoo et al., 2018; Perron et al., 2015), with additional code generated by Dr. Isaac Perron (python, https://pypi.org/project/eeg‐sleep‐analysis/).

### 4.4 Stereotaxic injection surgery

Stereotaxic hippocampal injections were performed to deliver a BiP viral overexpression vector (Vector Biolabs; AAV5‐CamKIIa‐GRP78; titer 4.8 × 10^12^ GC/ml) and an mCherry control vector (Addgene; pAAV5‐CaMKIIa‐mCherry; titer 2.3 × 10^−13^ GC/ml) into the hippocampi of young and aged mice. Stereotaxic injections were performed as described. Following general anesthesia (2% isoflurane) and sterilization of surgical area and tools, an incision was made on the top of the skull and local bupivacaine was administered. Four burr holes were drilled for bilateral hippocampal injections, with coordinates as follows: AP ±1.8 mm, ML ±0.8 mm, ±1.8 mm, DV −1.7 mm, −1.9 mm. Using a 1 µl Hamiliton Syringe, 50 nanoliters of virus were injected per burr hole. Mice were given subcutaneous injections of meloxicam and saline post‐operation for analgesia and hydration, respectively. Topical antibiotic ointment was used following the suturing of the surgical site. Mice were observed for 3 days of recovery. Behavioral testing occurred four weeks after surgery date to allow for sufficient viral expression. Transcardial perfusions were performed at ZT0 following testing, and half brains were collected and either flash frozen or fixed in 4% paraformaldehyde.

### 4.5 Y‐Maze spontaneous alternation test

The Y‐Maze test was performed as previously described (Kraeuter et al., 2019). Briefly, a single mouse was placed in the center of the apparatus and was allowed to move freely through the maze for 5 min. Each individual arm entry and the order in which the entries occurred were recorded. After testing, the number of alternations (3 separate arm sequential arm entries) was counted and presented as a percentage.

### 4.6 Spatial objection recognition (SOR) test

The spatial objection recognition test is well‐established hippocampal‐dependent spatial memory test (Bevins & Besheer, 2006; Cavoy & Delacour, 1993). The mice are placed together for an hour in the testing apparatus for three consecutive days prior to testing to acclimate to the container. Testing occurs in two phases. The first is the training phase where two identical objects are placed on one side of the apparatus. Mice are placed individually in the apparatus for 10 min and all interactions (smelling, touching, etc.) are counted. After training, the mice are returned to their home cage and left undisturbed for about one hour. Before the start of the testing phase, one of the identical objects is moved to the opposite side of the apparatus. Mice are placed in the apparatus during the testing phase similar to the training phase, but only for 3 minutes. Interactions with each object are measured again. Discrimination index calculations were performed as previously described as a measure for how well the mice distinguish between the moved and unmoved object (Sivakumaran et al., 2018).

### 4.7 Immunohistochemical assays

Post‐fixed half‐brain coronal sections were sliced at 40 μm using a cryostat as previously described (Zhu et al., 2007). Every other section was placed in 24‐well plates containing cryoprotectant for free‐floating immunohistochemistry staining and stored at −20ºC, as previously described (Naidoo et al., 2008, 2018). For all makers, we compared *n *= 5–8 in each of the four groups.

### 4.8 Immunofluorescence

Immunofluorescence (IF) staining was conducted as previously described (Naidoo et al., 2011). Primary antibodies are as follows: p‐CREB (ser133) (1:300, Cell Signaling 87G3); CREB (1:200, Cell Signaling 86B10); p‐PERK (Thr980) (1:200, Bioss bs‐3330R); ATF4 (1:500, ProteinTech, 60035‐1‐Ig); peIF2α (1:100, Cell Signaling 3597S); and BiP/anti‐KDEL (1:1000, Enzo Life Sciences ADI‐SPA‐827F). Secondary antibodies are as follows: Alexa Fluor 488 donkey anti‐rabbit IgG (1:500); Alex Flur 594 donkey anti‐mouse IgG (1:500); and Alexa Fluor 488 donkey anti‐mouse IgG (1:500).

### 4.9 Quantitative analysis of IF images

Confocal images were acquired as previously described (Owen et al., 2021), using Leica SP5/AOBS microscope. Confocal laser intensities, nm range, detector gain, exposure time, amplifier offset, and depth of the focal plane within sections per antigen target were standardized across compared sections. Confocal images were quantified as previously described (Zhu et al., 2018). Briefly, 3–4 sections were imaged per animal (*n* =4–5 animals per group). Using ImageJ software, the images were converted to an 8‐bit grayscale with detection threshold standardized across all images to detect percent areas. The percentage area covered within the target region was measured, and average percent areas for each mouse were analyzed.

### 4.10 Western blot staining

Frozen brain tissue was prepared for Western blot assays as previously described (Naidoo et al., 2008, 2018). Briefly, brain tissue was homogenized on ice with lysis buffer containing protease inhibitors. After centrifugation, protein concentration for each sample was determined with a BCA protein assay and samples were prepared such that each contained 20µg of protein. SDS‐PAGE gels were run as previously described (Naidoo et al., 2008), and protein bands were imaged and quantified via infrared imaging on an Odyssey scanner (LiCor). For all markers, we compared *n* = 5–8 for each of the groups. Primary antibodies are as follows: BiP/anti‐KDEL (1:1000, Enzo Life Sciences ADI‐SPA‐827F); GADD34 (1:500, Protein Tech 10449‐1‐AP); p‐AKT (1:500, Cell Signaling 9271); and Akt (1:500, Cell Signaling 9272). Secondary antibodies are as follows: LiCor IRDye 680RD Goat anti‐Mouse (1:10,000); LiCor IRDye 800RD Goat anti‐Mouse (1:10,000); LiCor IRDye 800RD Goat anti‐Rabbit (1:10,000); and Odyssey IRDye 680 Goat anti‐Rabbit (1:10,000).

### 4.11 Statistical analyses

Data are presented as the average ± standard error of the mean (SEM) of sample size *n*. Statistical analyses were performed in PRISM (GraphPad Software, La Jolla, CA). Unless otherwise specified, two‐way ANOVA was used to determine interaction effects, with Tukey post hoc corrections for multiple comparisons. *p *< 0.05 was the threshold for determining statistical significance.

## CONFLICT OF INTEREST

The authors have no conflicts to declare.

## AUTHOR'S CONTRIBUTIONS

JH designed and conducted the experiments, analyzed data, and wrote the manuscript. ES performed biochemical assays. NN was involved in study design, data interpretation, and writing and editing of the manuscript.

## Supporting information

Supplementary Material

## Data Availability

The raw data supporting the conclusions in this manuscript will be made available upon request.

## References

[acel13598-bib-0001] Alexandre, C. , Andermann, M. L. , & Scammell, T. E. (2013). Control of arousal by the orexin neurons. Current Opinion in Neurobiology, 23, 752–759. 10.1016/j.conb.2013.04.008 23683477 PMC3783629

[acel13598-bib-0002] Aton, S. J. , Seibt, J. , Dumoulin, M. , Jha, S. K. , Steinmetz, N. , Coleman, T. , Naidoo, N. , & Frank, M. G. (2009). Mechanisms of sleep‐dependent consolidation of cortical plasticity. Neuron, 61, 454–466. 10.1016/j.neuron.2009.01.007 19217381 PMC2665998

[acel13598-bib-0003] Bekinschtein, P. , Cammarota, M. , Katche, C. , Slipczuk, L. , Rossato, J. I. , Goldin, A. , Izquierdo, I. , & Medina, J. H. (2008). BDNF is essential to promote persistence of long‐term memory storage. Proceedings of the National Academy of Sciences of the United States of America, 105, 2711–2716. 10.1073/pnas.0711863105 18263738 PMC2268201

[acel13598-bib-0004] Berridge, M. J. (2002). The endoplasmic reticulum: a multifunctional signaling organelle. Cell Calcium, 32, 235–249. 10.1016/S0143416002001823 12543086

[acel13598-bib-0005] Bevins, R. A. , & Besheer, J. (2006). Object recognition in rats and mice: a one‐trial non‐matching‐to‐sample learning task to study ‘recognition memory'. Nature Protocols, 1, 1306–1311. 10.1038/nprot.2006.205 17406415

[acel13598-bib-0006] Blendy, J. A. , Kaestner, K. H. , Schmid, W. , Gass, P. , & Schutz, G. (1996). Targeting of the CREB gene leads to up‐regulation of a novel CREB mRNA isoform. The EMBO Journal, 15, 1098–1106. 10.1002/j.1460-2075.1996.tb00447.x 8605879 PMC450007

[acel13598-bib-0007] Bourtchuladze, R. , Frenguelli, B. , Blendy, J. , Cioffi, D. , Schutz, G. , & Silva, A. J. (1994). Deficient long‐term memory in mice with a targeted mutation of the cAMP‐responsive element‐binding protein. Cell, 79, 59–68. 10.1016/0092-8674(94)90400-6 7923378

[acel13598-bib-0008] Brown, M. K. , Chan, M. T. , Zimmerman, J. E. , Pack, A. I. , Jackson, N. E. , & Naidoo, N. (2014). Aging induced endoplasmic reticulum stress alters sleep and sleep homeostasis. Neurobiology of Aging, 35, 1431–1441. 10.1016/j.neurobiolaging.2013.12.005 24444805 PMC4019391

[acel13598-bib-0009] Brown, M. K. , & Naidoo, N. (2012). The endoplasmic reticulum stress response in aging and age‐related diseases. Frontiers in Physiology, 3, 263. 10.3389/fphys.2012.00263 22934019 PMC3429039

[acel13598-bib-0010] Cao, A. L. , Wang, L. , Chen, X. , Wang, Y. M. , Guo, H. J. , Chu, S. , Liu, C. , Zhang, X. M. , & Peng, W. (2016). Ursodeoxycholic acid and 4‐phenylbutyrate prevent endoplasmic reticulum stress‐induced podocyte apoptosis in diabetic nephropathy. Laboratory Investigation; a Journal of Technical Methods and Pathology, 96, 610–622. 10.1038/labinvest.2016.44 26999661

[acel13598-bib-0011] Cavoy, A. , & Delacour, J. (1993). Spatial but not object recognition is impaired by aging in rats. Physiology & Behavior, 53, 527–530. 10.1016/0031-9384(93)90148-9 8451318

[acel13598-bib-0012] Chellappa, K. , Perron, I. J. , Naidoo, N. , & Baur, J. A. (2019). The leptin sensitizer celastrol reduces age‐associated obesity and modulates behavioral rhythms. Aging Cell, 18, e12874. 10.1111/acel.12874 30821426 PMC6516176

[acel13598-bib-0013] Chuluun, B. , Pittaras, E. , Hong, H. , Fisher, N. , Colas, D. , Ruby, N. F. , & Heller, H. C. (2020). Suprachiasmatic lesions restore object recognition in down syndrome model mice. Neurobiology of Sleep and Circadian Rhythms, 8, 100049. 10.1016/j.nbscr.2020.100049 32195448 PMC7075983

[acel13598-bib-0014] Cirelli, C. , & Tononi, G. (2000). Gene expression in the brain across the sleep‐waking cycle. Brain Research, 885, 303–321. 11102586 10.1016/s0006-8993(00)03008-0

[acel13598-bib-0015] Costa‐Mattioli, M. , Gobert, D. , Stern, E. , Gamache, K. , Colina, R. , Cuello, C. , Sossin, W. , Kaufman, R. , Pelletier, J. , Rosenblum, K. , Krnjevic, K. , Lacaille, J. C. , Nader, K. , & Sonenberg, N. (2007). eIF2alpha phosphorylation bidirectionally regulates the switch from short‐ to long‐term synaptic plasticity and memory. Cell, 129, 195–206. 17418795 10.1016/j.cell.2007.01.050 PMC4149214

[acel13598-bib-0016] Diekelmann, S. , & Born, J. (2010). The memory function of sleep. Nature Reviews Neuroscience, 11, 114. 10.1038/nrn2762 20046194

[acel13598-bib-0017] Du, K. , & Montminy, M. (1998). CREB is a regulatory target for the protein kinase Akt/PKB. The Journal of Biological Chemistry, 273, 32377–32379. 10.1074/jbc.273.49.32377 9829964

[acel13598-bib-0018] Farook, J. M. , Shields, J. , Tawfik, A. , Markand, S. , Sen, T. , Smith, S. B. , Brann, D. , Dhandapani, K. M. , & Sen, N. (2013). GADD34 induces cell death through inactivation of Akt following traumatic brain injury. Cell Death & Disease, 4, e754. 10.1038/cddis.2013.280 23907468 PMC3763442

[acel13598-bib-0019] Franken, P. , Chollet, D. , & Tafti, M. (2001). The homeostatic regulation of sleep need is under genetic control. The Journal of Neuroscience: the Official Journal of the Society for Neuroscience, 21, 2610–2621. 10.1523/JNEUROSCI.21-08-02610.2001 11306614 PMC6762509

[acel13598-bib-0020] Gonzalez, M. C. , Radiske, A. , & Cammarota, M. (2019). On the involvement of BDNF signaling in memory reconsolidation. Frontiers in Cellular Neuroscience, 13, 383. 10.3389/fncel.2019.00383 31507380 PMC6713924

[acel13598-bib-0021] Graves, L. A. , Hellman, K. , Veasey, S. , Blendy, J. A. , Pack, A. I. , & Abel, T. (2003). Genetic evidence for a role of CREB in sustained cortical arousal. Journal of Neurophysiology, 90, 1152–1159. 10.1152/jn.00882.2002 12711709

[acel13598-bib-0022] Gulia, K. K. , & Kumar, V. M. (2018). Sleep disorders in the elderly: a growing challenge. Psychogeriatrics: the Official Journal of the Japanese Psychogeriatric Society, 18, 155–165. 10.1111/psyg.12319 29878472

[acel13598-bib-0023] Guo, Q. , Xu, L. , Li, H. , Sun, H. , Wu, S. , & Zhou, B. (2017). 4‐PBA reverses autophagic dysfunction and improves insulin sensitivity in adipose tissue of obese mice via Akt/mTOR signaling. Biochemical and Biophysical Research Communications, 484, 529–535. 10.1016/j.bbrc.2017.01.106 28153729

[acel13598-bib-0024] Haettig, J. , Stefanko, D. P. , Multani, M. L. , Figueroa, D. X. , McQuown, S. C. , & Wood, M. A. (2011). HDAC inhibition modulates hippocampus‐dependent long‐term memory for object location in a CBP‐dependent manner. Learning & Memory, 18, 71–79. 10.1101/lm.1986911 21224411 PMC3032579

[acel13598-bib-0025] Halliday, M. , & Mallucci, G. R. (2014). Targeting the unfolded protein response in neurodegeneration: A new approach to therapy. Neuropharmacology, 76(Pt A), 169–174. 10.1016/j.neuropharm.2013.08.034 24035917

[acel13598-bib-0026] Halliday, M. , & Mallucci, G. R. (2015). Review: Modulating the unfolded protein response to prevent neurodegeneration and enhance memory. Neuropathology and Applied Neurobiology, 41, 414–427. 10.1111/nan.12211 25556298 PMC5053297

[acel13598-bib-0027] Halliday, M. , Radford, H. , Sekine, Y. , Moreno, J. , Verity, N. , le Quesne, J. , Ortori, C. A. , Barrett, D. A. , Fromont, C. , Fischer, P. M. , Harding, H. P. , Ron, D. , & Mallucci, G. R. (2015). Partial restoration of protein synthesis rates by the small molecule ISRIB prevents neurodegeneration without pancreatic toxicity. Cell Death & Disease, 6, e1672. 10.1038/cddis.2015.49 25741597 PMC4385927

[acel13598-bib-0028] Hasan, S. , Dauvilliers, Y. , Mongrain, V. , Franken, P. , & Tafti, M. (2012). Age‐related changes in sleep in inbred mice are genotype dependent. Neurobiology of Aging, 33(195), e113–126. 10.1016/j.neurobiolaging.2010.05.010 20619936

[acel13598-bib-0029] Havekes, R. , Vecsey, C. G. , & Abel, T. (2012). The impact of sleep deprivation on neuronal and glial signaling pathways important for memory and synaptic plasticity. Cellular Signalling, 24, 1251–1260. 10.1016/j.cellsig.2012.02.010 22570866 PMC3622220

[acel13598-bib-0030] Helfrich, R. F. , Mander, B. A. , Jagust, W. J. , Knight, R. T. , & Walker, M. P. (2018). Old brains come uncoupled in sleep: Slow wave‐spindle synchrony, brain atrophy, and forgetting. Neuron, 97, 221–230.e4. 10.1016/j.neuron.2017.11.020 29249289 PMC5754239

[acel13598-bib-0031] Hernandez, P. J. , & Abel, T. (2008). The role of protein synthesis in memory consolidation: progress amid decades of debate. Neurobiology of Learning and Memory, 89, 293–311. 10.1016/j.nlm.2007.09.010 18053752 PMC2745628

[acel13598-bib-0032] Hetz, C. , & Mollereau, B. (2014). Disturbance of endoplasmic reticulum proteostasis in neurodegenerative diseases. Nature Reviews. Neuroscience, 15, 233–249. 10.1038/nrn3689 24619348

[acel13598-bib-0033] Hetz, C. , Zhang, K. , & Kaufman, R. J. (2020). Mechanisms, regulation and functions of the unfolded protein response. Nature Reviews. Molecular Cell Biology, 21, 421–438. 10.1038/s41580-020-0250-z 32457508 PMC8867924

[acel13598-bib-0034] Hughes, D. , & Mallucci, G. R. (2019). The unfolded protein response in neurodegenerative disorders – therapeutic modulation of the PERK pathway. The FEBS Journal, 286, 342–355. 10.1111/febs.14422 29476642

[acel13598-bib-0035] Hussain, S. G. , & Ramaiah, K. V. (2007). Reduced eIF2alpha phosphorylation and increased proapoptotic proteins in aging. Biochemical and Biophysical Research Communications, 355, 365–370. 17300747 10.1016/j.bbrc.2007.01.156

[acel13598-bib-0036] Kanasi, E. , Ayilavarapu, S. , & Jones, J. (2016). The aging population: demographics and the biology of aging. Periodontology, 2000(72), 13–18. 10.1111/prd.12126 27501488

[acel13598-bib-0037] Kaufman, R. J. (2002). Orchestrating the unfolded protein response in health and disease. The Journal of Clinical Investigation, 110, 1389–1398. 10.1172/JCI0216886 12438434 PMC151822

[acel13598-bib-0038] Kim, J. , Park, H. , Im, J. Y. , Choi, W. S. , & Kim, H. S. (2007). Sodium butyrate regulates androgen receptor expression and cell cycle arrest in human prostate cancer cells. Anticancer Research, 27, 3285–3292. 17970072

[acel13598-bib-0039] Koga, H. , Kaushik, S. , & Cuervo, A. M. (2011). Protein homeostasis and aging: The importance of exquisite quality control. Ageing Research Reviews, 10, 205–215. 10.1016/j.arr.2010.02.001 20152936 PMC2888802

[acel13598-bib-0040] Koh, K. , Evans, J. M. , Hendricks, J. C. , & Sehgal, A. (2006). A Drosophila model for age‐associated changes in sleep:wake cycles. Proceedings of the National Academy of Sciences of the United States of America, 103, 13843–13847. 16938867 10.1073/pnas.0605903103 PMC1564207

[acel13598-bib-0041] Kraeuter, A. K. , Guest, P. C. , & Sarnyai, Z. (2019). The Y‐Maze for Assessment of Spatial Working and Reference Memory in Mice. Methods in Molecular Biology, 1916, 105–111. 30535688 10.1007/978-1-4939-8994-2_10

[acel13598-bib-0042] Krone, L. B. , Yamagata, T. , Blanco‐Duque, C. , Guillaumin, M. C. C. , Kahn, M. C. , van der Vinne, V. , McKillop, L. E. , Tam, S. K. E. , Peirson, S. N. , Akerman, C. J. , Hoerder‐Suabedissen, A. , Molnar, Z. , & Vyazovskiy, V. V. (2021). A role for the cortex in sleep‐wake regulation. Nature Neuroscience, 24, 1210–1215. 10.1038/s41593-021-00894-6 34341585 PMC7612118

[acel13598-bib-0043] Kumar, A. (2011). Long‐Term Potentiation at CA3‐CA1 Hippocampal Synapses with Special Emphasis on Aging, Disease, and Stress. Frontiers in Aging Neuroscience, 3, 7. 10.3389/fnagi.2011.00007 21647396 PMC3102214

[acel13598-bib-0044] Lee, K. , Tirasophon, W. , Shen, X. , Michalak, M. , Prywes, R. , Okada, T. , Yoshida, H. , Mori, K. , & Kaufman, R. J. (2002). IRE1‐mediated unconventional mRNA splicing and S2P‐mediated ATF6 cleavage merge to regulate XBP1 in signaling the unfolded protein response. Genes & Development, 16, 452–466. 10.1101/gad.964702 11850408 PMC155339

[acel13598-bib-0045] Li, H. , Wen, W. , Xu, H. , Wu, H. , Xu, M. , Frank, J. A. , & Luo, J. (2019). 4‐Phenylbutyric acid protects against ethanol‐induced damage in the developing mouse brain. Alcoholism, Clinical and Experimental Research, 43, 69–78. 30403409 10.1111/acer.13918 PMC6318008

[acel13598-bib-0046] Lim, M. M. , Elkind, J. , Xiong, G. , Galante, R. , Zhu, J. , Zhang, L. , Lian, J. , Rodin, J. , Kuzma, N. N. , Pack, A. I. , & Cohen, A. S. (2013). Dietary therapy mitigates persistent wake deficits caused by mild traumatic brain injury. Science Translational Medicine, 5, 215ra173. 10.1126/scitranslmed.3007092 PMC3951738 24337480

[acel13598-bib-0047] Lisman, J. E. (1999). Relating hippocampal circuitry to function: recall of memory sequences by reciprocal dentate‐CA3 interactions. Neuron, 22, 233–242. 10.1016/S0896-6273(00)81085-5 10069330

[acel13598-bib-0048] Ly, S. , Lee, D. A. , Strus, E. , Prober, D. A. , & Naidoo, N. (2020). Evolutionarily conserved regulation of sleep by the protein translational regulator PERK. Current Biology, 30(9), 1639–1648.e3. 10.1016/j.cub.2020.02.030 32169212 PMC8788386

[acel13598-bib-0049] Ma, T. , Trinh, M. A. , Wexler, A. J. , Bourbon, C. , Gatti, E. , Pierre, P. , Cavener, D. R. , & Klann, E. (2013). Suppression of eIF2alpha kinases alleviates Alzheimer's disease‐related plasticity and memory deficits. Nature Neuroscience, 16, 1299–1305. 23933749 10.1038/nn.3486 PMC3756900

[acel13598-bib-0050] Mander, B. A. , Winer, J. R. , & Walker, M. P. (2017). Sleep and human aging. Neuron, 94, 19–36. 10.1016/j.neuron.2017.02.004 28384471 PMC5810920

[acel13598-bib-0051] Mendelson, W. B. , & Bergmann, B. M. (1999). Age‐related changes in sleep in the rat. Sleep, 22, 145–150. 10.1093/sleep/22.2.145 10201059

[acel13598-bib-0052] Methippara, M. M. , Bashir, T. , Kumar, S. , Alam, N. , Szymusiak, R. , & McGinty, D. (2009). Salubrinal, an inhibitor of protein synthesis, promotes deep slow wave sleep. Am J Physiol Regul Integr Comp Physiol, 296(1), 178–84. 10.1016/j.neuroscience.2007.09.051 PMC2636978 18971348

[acel13598-bib-0053] Mochizuki, T. , Crocker, A. , McCormack, S. , Yanagisawa, M. , Sakurai, T. , & Scammell, T. E. (2004). Behavioral state instability in orexin knock‐out mice. The Journal of Neuroscience : the Official Journal of the Society for Neuroscience, 24, 6291–6300. 10.1523/JNEUROSCI.0586-04.2004 15254084 PMC6729542

[acel13598-bib-0054] Moreno, J. A. , Radford, H. , Peretti, D. , Steinert, J. R. , Verity, N. , Martin, M. G. , Halliday, M. , Morgan, J. , Dinsdale, D. , Ortori, C. A. , Barrett, D. A. , Tsaytler, P. , Bertolotti, A. , Willis, A. E. , Bushell, M. , & Mallucci, G. R. (2012). Sustained translational repression by eIF2alpha‐P mediates prion neurodegeneration. Nature, 485, 507–511. 22622579 10.1038/nature11058 PMC3378208

[acel13598-bib-0055] Muzur, A. , Pace‐Schott, E. F. , & Hobson, J. A. (2002). The prefrontal cortex in sleep. Trends in Cognitive Sciences, 6, 475–481. 10.1016/S1364-6613(02)01992-7 12457899

[acel13598-bib-0057] Naidoo, N. , Casiano, V. , Cater, J. , Zimmerman, J. , & Pack, A. I. (2007). A role for the molecular chaperone protein BiP/GRP78 in Drosophila sleep homeostasis. Sleep, 30, 557–565. 10.1093/sleep/30.5.557 17552370

[acel13598-bib-0058] Naidoo, N. , Ferber, M. , Master, M. , Zhu, Y. , & Pack, A. I. (2008). Aging impairs the unfolded protein response to sleep deprivation and leads to proapoptotic signaling. The Journal of Neuroscience: the Official Journal of the Society for Neuroscience, 28, 6539–6548. 10.1523/JNEUROSCI.5685-07.2008 18579727 PMC2925257

[acel13598-bib-0059] Naidoo, N. , Giang, W. , Galante, R. J. , & Pack, A. I. (2005). Sleep deprivation induces the unfolded protein response in mouse cerebral cortex. Journal of Neurochemistry, 92, 1150–1157. 10.1111/j.1471-4159.2004.02952.x 15715665

[acel13598-bib-0060] Naidoo, N. , Zhu, J. , Galante, R. J. , Lian, J. , Strus, E. , Lee, A. , Keenan, B. T. , & Pack, A. I. (2018). Reduction of the molecular chaperone binding immunoglobulin protein (BiP) accentuates the effect of aging on sleep‐wake behavior. Neurobiology of Aging, 69, 10–25. 10.1016/j.neurobiolaging.2018.04.011 29843048 PMC6064380

[acel13598-bib-0061] Naidoo, N. , Zhu, J. , Zhu, Y. , Fenik, P. , Lian, J. , Galante, R. , & Veasey, S. (2011). Endoplasmic reticulum stress in wake‐active neurons progresses with aging. Aging Cell, 10, 640–649. 10.1111/j.1474-9726.2011.00699.x 21388495 PMC3125474

[acel13598-bib-0062] National Institute on Aging. N.I.o.H. (2007). Why population aging matters: A global perspective. National Institute on Aging. N.I.o.H.

[acel13598-bib-0063] Nebes, R. D. , Buysse, D. J. , Halligan, E. M. , Houck, P. R. , & Monk, T. H. (2009). Self‐reported sleep quality predicts poor cognitive performance in healthy older adults. The Journals of Gerontology. Series B, Psychological Sciences and Social Sciences, 64, 180–187. 10.1093/geronb/gbn037 19204069 PMC2655169

[acel13598-bib-0064] Ohayon, M. M. , & Vecchierini, M. F. (2002). Daytime sleepiness and cognitive impairment in the elderly population. Archives of Internal Medicine, 162, 201–208. 10.1001/archinte.162.2.201 11802754

[acel13598-bib-0065] Owen, J. E. , Zhu, Y. , Fenik, P. , Zhan, G. , Bell, P. , Liu, C. , & Veasey, S. (2021). Late‐in‐life neurodegeneration after chronic sleep loss in young adult mice. Sleep, 44, zsab057. 10.1093/sleep/zsab057 33768250 PMC8361366

[acel13598-bib-0066] Ozcan, U. , Yilmaz, E. , Ozcan, L. , Furuhashi, M. , Vaillancourt, E. , Smith, R. O. , Gorgun, C. Z. , & Hotamisligil, G. S. (2006). Chemical chaperones reduce ER stress and restore glucose homeostasis in a mouse model of type 2 diabetes. Science, 313, 1137–1140. 10.1126/science.1128294 16931765 PMC4741373

[acel13598-bib-0067] Pandi‐Perumal, S. R. , Seils, L. K. , Kayumov, L. , Ralph, M. R. , Lowe, A. , Moller, H. , & Swaab, D. F. (2002). Senescence, sleep, and circadian rhythms. Ageing Research Reviews, 1, 559–604. 10.1016/S1568-1637(02)00014-4 12067601

[acel13598-bib-0068] Panja, D. , & Bramham, C. R. (2014). BDNF mechanisms in late LTP formation: A synthesis and breakdown. Neuropharmacology, 76(Pt C), 664–676. 10.1016/j.neuropharm.2013.06.024 23831365

[acel13598-bib-0069] Paz Gavilan, M. , Vela, J. , Castano, A. , Ramos, B. , del Rio, J. C. , Vitorica, J. , & Ruano, D. (2006). Cellular environment facilitates protein accumulation in aged rat hippocampus. Neurobiology of Aging, 27, 973–982. 10.1016/j.neurobiolaging.2005.05.010 15964666

[acel13598-bib-0070] Perron, I. J. , Pack, A. I. , & Veasey, S. (2015). Diet/Energy balance affect sleep and wakefulness independent of body weight. Sleep, 38, 1893–1903. 10.5665/sleep.5236 26158893 PMC4667395

[acel13598-bib-0071] Radford, H. , Moreno, J. A. , Verity, N. , Halliday, M. , & Mallucci, G. R. (2015). PERK inhibition prevents tau‐mediated neurodegeneration in a mouse model of frontotemporal dementia. Acta Neuropathologica, 130, 633–642. 10.1007/s00401-015-1487-z 26450683 PMC4612323

[acel13598-bib-0072] Ricobaraza, A. , Cuadrado‐Tejedor, M. , & Garcia‐Osta, A. (2011). Long‐term phenylbutyrate administration prevents memory deficits in Tg2576 mice by decreasing Abeta. Frontiers in Bioscience (Elite Ed), 3, 1375–1384. 10.2741/e340 21622143

[acel13598-bib-0073] Ricobaraza, A. , Cuadrado‐Tejedor, M. , Pérez‐Mediavilla, A. , Frechilla, D. , Del Río, J. , & García‐Osta, A. (2009). Phenylbutyrate ameliorates cognitive deficit and reduces tau pathology in an Alzheimer's disease mouse model. Neuropsychopharmacology, 34, 1721. 19145227 10.1038/npp.2008.229

[acel13598-bib-0074] Ron, D. , & Walter, P. (2007). Signal integration in the endoplasmic reticulum unfolded protein response. Nature Reviews. Molecular Cell Biology, 8, 519–529. 10.1038/nrm2199 17565364

[acel13598-bib-0075] Schmutte, T. , Harris, S. , Levin, R. , Zweig, R. , Katz, M. , & Lipton, R. (2007). The relation between cognitive functioning and self‐reported sleep complaints in nondemented older adults: results from the Bronx aging study. Behavioral Sleep Medicine, 5, 39–56. 10.1207/s15402010bsm0501_3 17313323

[acel13598-bib-0076] Seibt, J. , Dumoulin, M. C. , Aton, S. J. , Coleman, T. , Watson, A. , Naidoo, N. , & Frank, M. G. (2012). Protein synthesis during sleep consolidates cortical plasticity in vivo. Current Biology: CB, 22, 676–682. 22386312 10.1016/j.cub.2012.02.016 PMC3350782

[acel13598-bib-0077] Sen, N. (2019). ER stress, CREB, and memory: A tangled emerging link in disease. The Neuroscientist: a Review Journal Bringing Neurobiology, Neurology and Psychiatry, 25, 420–433. 10.1177/1073858418816611 30477403 PMC6535379

[acel13598-bib-0078] Sen, T. , Gupta, R. , Kaiser, H. , & Sen, N. (2017). Activation of PERK elicits memory impairment through inactivation of CREB and downregulation of PSD95 After traumatic brain injury. The Journal of Neuroscience: the Official Journal of the Society for Neuroscience, 37, 5900–5911. 10.1523/JNEUROSCI.2343-16.2017 28522733 PMC5473207

[acel13598-bib-0079] Sharma, R. , Sahota, P. , & Thakkar, M. M. (2021). Short‐term sleep deprivation immediately after contextual conditioning inhibits BDNF signaling and disrupts memory consolidation in predator odor trauma mice model of PTSD. Brain Research, 1750, 147155. 10.1016/j.brainres.2020.147155 33069732

[acel13598-bib-0080] Sharma, V. , Ounallah‐Saad, H. , Chakraborty, D. , Hleihil, M. , Sood, R. , Barrera, I. , Edry, E. , Kolatt Chandran, S. , Ben Tabou de Leon, S. , Kaphzan, H. , & Rosenblum, K. (2018). Local inhibition of PERK enhances memory and reverses age‐related deterioration of cognitive and neuronal properties. The Journal of Neuroscience: the Official Journal of the Society for Neuroscience, 38, 648–658. 10.1523/JNEUROSCI.0628-17.2017 29196323 PMC6596193

[acel13598-bib-0081] Sivakumaran, M. H. , Mackenzie, A. K. , Callan, I. R. , Ainge, J. A. , & O'Connor, A. R. (2018). The discrimination ratio derived from novel object recognition tasks as a measure of recognition memory sensitivity, not bias. Scientific Reports, 8, 11579. 10.1038/s41598-018-30030-7 30069031 PMC6070491

[acel13598-bib-0082] Soltani, S. , Chauvette, S. , Bukhtiyarova, O. , Lina, J. M. , Dube, J. , Seigneur, J. , Carrier, J. , & Timofeev, I. (2019). Sleep‐wake cycle in young and older mice. Frontiers in Systems Neuroscience, 13, 51. 10.3389/fnsys.2019.00051 31611779 PMC6769075

[acel13598-bib-0083] Szegezdi, E. , Logue, S. E. , Gorman, A. M. , & Samali, A. (2006). Mediators of endoplasmic reticulum stress‐induced apoptosis. EMBO Reports, 7, 880–885. 10.1038/sj.embor.7400779 16953201 PMC1559676

[acel13598-bib-0084] Taylor, R. C. , & Dillin, A. (2013). XBP‐1 is a cell‐nonautonomous regulator of stress resistance and longevity. Cell, 153, 1435–1447. 10.1016/j.cell.2013.05.042 23791175 PMC4771415

[acel13598-bib-0085] Tononi, G. , & Cirelli, C. (2003). Sleep and synaptic homeostasis: a hypothesis. Brain Research Bulletin, 62, 143–150. 10.1016/j.brainresbull.2003.09.004 14638388

[acel13598-bib-0086] Tsaytler, P. , & Bertolotti, A. (2013). Exploiting the selectivity of protein phosphatase 1 for pharmacological intervention. FEBS Journal, 280, 766–770. 10.1111/j.1742-4658.2012.08535.x 22340633

[acel13598-bib-0087] Tudor, J. C. , Davis, E. J. , Peixoto, L. , Wimmer, M. E. , van Tilborg, E. , Park, A. J. , Poplawski, S. G. , Chung, C. W. , Havekes, R. , Huang, J. , Gatti, E. , Pierre, P. , & Abel, T. (2016). Sleep deprivation impairs memory by attenuating mTORC1‐dependent protein synthesis. Science Signaling, 9, ra41. 10.1126/scisignal.aad4949 27117251 PMC4890572

[acel13598-bib-0088] United Nations, D.o.E.a.S.A., Population Division (2017). World Population Ageing 2017 ‐ Highlights In (ST/ESA/SER.A/397).

[acel13598-bib-0089] Vienne, J. , Spann, R. , Guo, F. , & Rosbash, M. (2016). Age‐related reduction of recovery sleep and arousal threshold in drosophila. Sleep, 39, 1613–1624. 27306274 10.5665/sleep.6032 PMC4945321

[acel13598-bib-0090] Welsh, D. K. , Richardson, G. S. , & Dement, W. C. (1986). Effect of age on the circadian pattern of sleep and wakefulness in the mouse. Journal of Gerontology, 41, 579–586. 10.1093/geronj/41.5.579 3745812

[acel13598-bib-0091] Wimmer, M. E. , Hernandez, P. J. , Blackwell, J. , & Abel, T. (2012). Aging impairs hippocampus‐dependent long‐term memory for object location in mice. Neurobiology of Aging, 33, 2220–2224. 10.1016/j.neurobiolaging.2011.07.007 21872364 PMC3227775

[acel13598-bib-0092] Wimmer, M. E. , Rising, J. , Galante, R. J. , Wyner, A. , Pack, A. I. , & Abel, T. (2013). Aging in mice reduces the ability to sustain sleep/wake states. PLoS One, 8, e81880. 10.1371/journal.pone.0081880 24358130 PMC3864844

[acel13598-bib-0093] Wolkove, N. , Elkholy, O. , Baltzan, M. , & Palayew, M. (2007). Sleep and aging: 1. Sleep disorders commonly found in older people. CMAJ: Canadian Medical Association Journal, 176, 1299–1304. 17452665 10.1503/cmaj.060792 PMC1852874

[acel13598-bib-0094] Xiong, W. X. , Zhou, G. X. , Wang, B. , Xue, Z. G. , Wang, L. , Sun, H. C. , & Ge, S. J. (2013). Impaired spatial learning and memory after sevoflurane‐nitrous oxide anesthesia in aged rats is associated with down‐regulated cAMP/CREB signaling. PLoS One, 8, e79408. 10.1371/journal.pone.0079408 24260214 PMC3829840

[acel13598-bib-0095] Yu, X. W. , Curlik, D. M. , Oh, M. M. , Yin, J. C. , & Disterhoft, J. F. (2017). CREB overexpression in dorsal CA1 ameliorates long‐term memory deficits in aged rats. eLife, 6, e19358. 10.7554/eLife.19358 28051768 PMC5214885

[acel13598-bib-0096] Zhang, T. , Ding, C. , Zhao, M. , Dai, X. , Yang, J. , Li, Y. , Gu, L. , Wei, Y. , Gong, J. , Zhu, W. , Li, N. , & Li, J. (2016). Sodium butyrate reduces colitogenic immunoglobulin a‐coated bacteria and modifies the composition of microbiota in IL‐10 deficient mice. Nutrients, 8, 728. 10.3390/nu8120728 PMC5188405 27886121

[acel13598-bib-0097] Zhou, G. , Xiong, W. , Zhang, X. , & Ge, S. (2013). Retrieval of consolidated spatial memory in the water maze is correlated with expression of pCREB and Egr1 in the hippocampus of aged mice. Dementia and Geriatric Cognitive Disorders Extra, 3, 39–47. 10.1159/000348349 23569457 PMC3618049

[acel13598-bib-0098] Zhu, Y. , Fenik, P. , Zhan, G. , Mazza, E. , Kelz, M. , Aston‐Jones, G. , & Veasey, S. C. (2007). Selective loss of catecholaminergic wake active neurons in a murine sleep apnea model. The Journal of Neuroscience: the Official Journal of the Society for Neuroscience, 27, 10060–10071. 10.1523/JNEUROSCI.0857-07.2007 17855620 PMC6672651

[acel13598-bib-0099] Zhu, Y. , Zhan, G. , Fenik, P. , Brandes, M. , Bell, P. , Francois, N. , Shulman, K. , & Veasey, S. (2018). Chronic sleep disruption advances the temporal progression of tauopathy in P301S mutant mice. The Journal of Neuroscience: the Official Journal of the Society for Neuroscience, 38, 10255–10270. 10.1523/JNEUROSCI.0275-18.2018 30322903 PMC6262148

