# Supplementary material for: Reducing ER stress with chaperone therapy reverses sleep fragmentation and cognitive decline in aged mice
Source: Aging Cell. 2022 Apr 30;21(6):e13598. doi: 10.1111/acel.13598 (PMC9197403; doi:10.1111/acel.13598)
Supplement: Supplementary file 1 — Supplementary Material [file ACEL-21-e13598-s001.docx]

**Supplemental Figures**


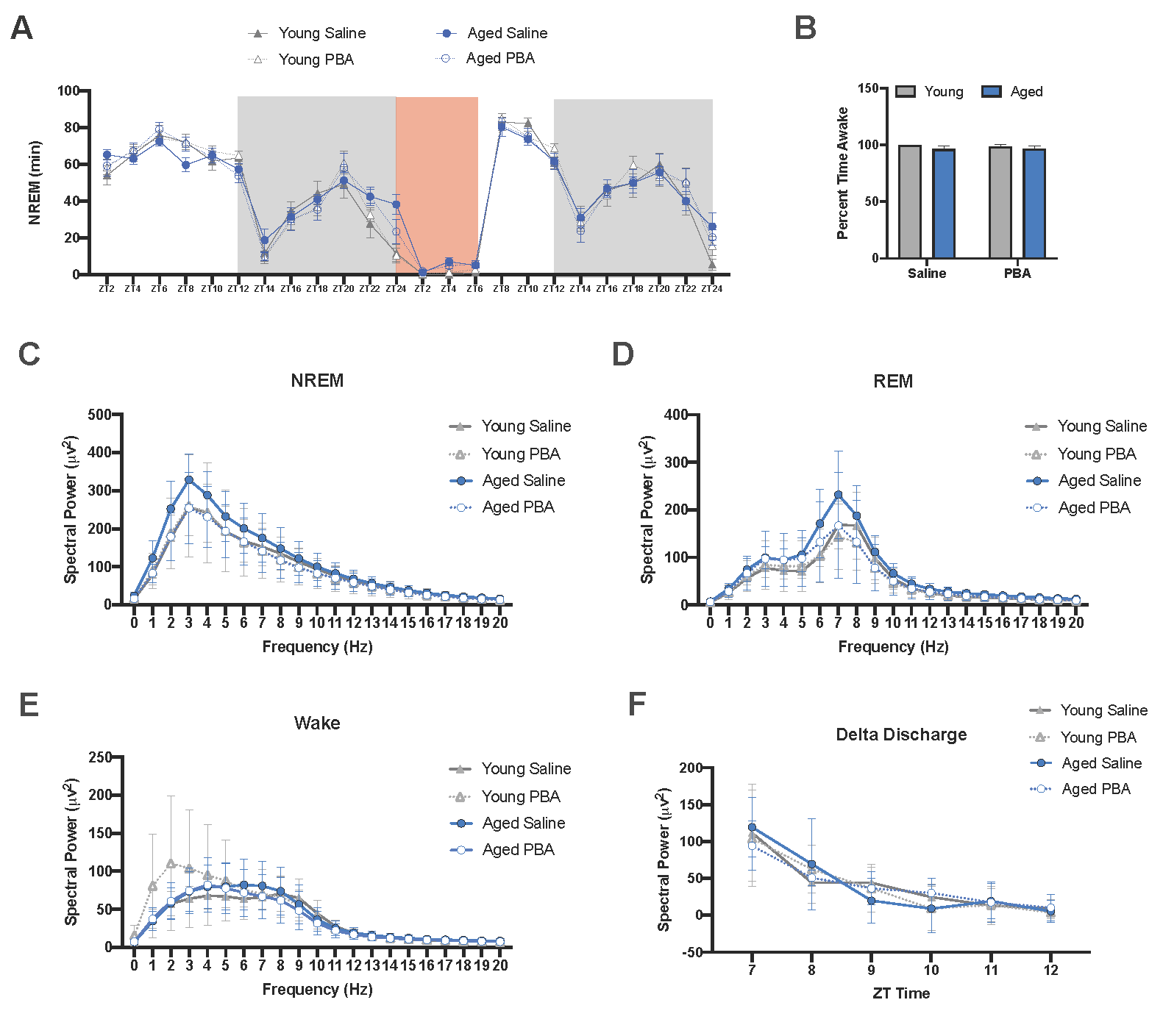


Supplemental Figure 1: Chaperone treatment does not alter spectral power density in either young or aged mice; **A)** Representative trace of the EEG recording scheme showing total NREM sleep averages for all four groups of mice across the 48-hr recording time. Gray indicates lights off (active phase) and red indicates 6hr sleep deprivation. **B)** Quantification examining percent time awake during 6hrs of sleep deprivation. **C-E)** Spectral power density was examined during the first 24hrs of recordings **C)** Spectrogram of NREM during inactive lights on period, **D)** Spectrogram of REM during inactive lights on period, **E)** Spectrogram of wake during active lights off period, **F)** Delta power discharge following sleep deprivation; delta power during recovery sleep (day 2 of recordings, ZT6-12) was compared to delta power during the corresponding baseline ZT time; (n = 5-6 for all groups, data presented as average power ± SEM).


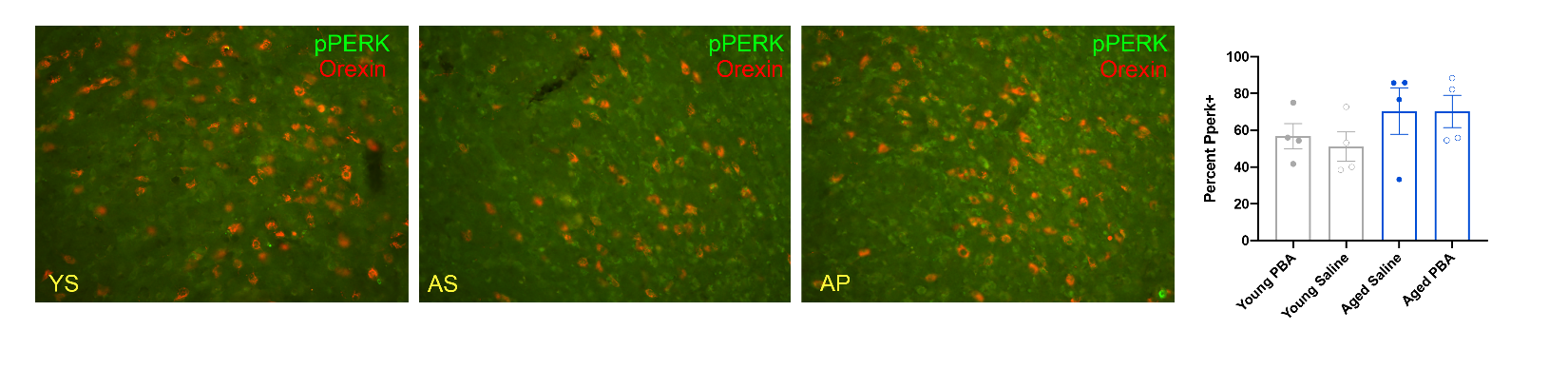


Supplemental Figure 2: p-PERK colocalization in orexin neurons with and without chaperone treatment. Representative images of pPERK and orexin double stains for three groups (n=4 animals per group). Graph quantifying percentage of orexin+ cells that were also pPERK+. Data is presented as average ± SEM. (Abbrev YS = young saline, AS = aged saline, AP = aged PBA).


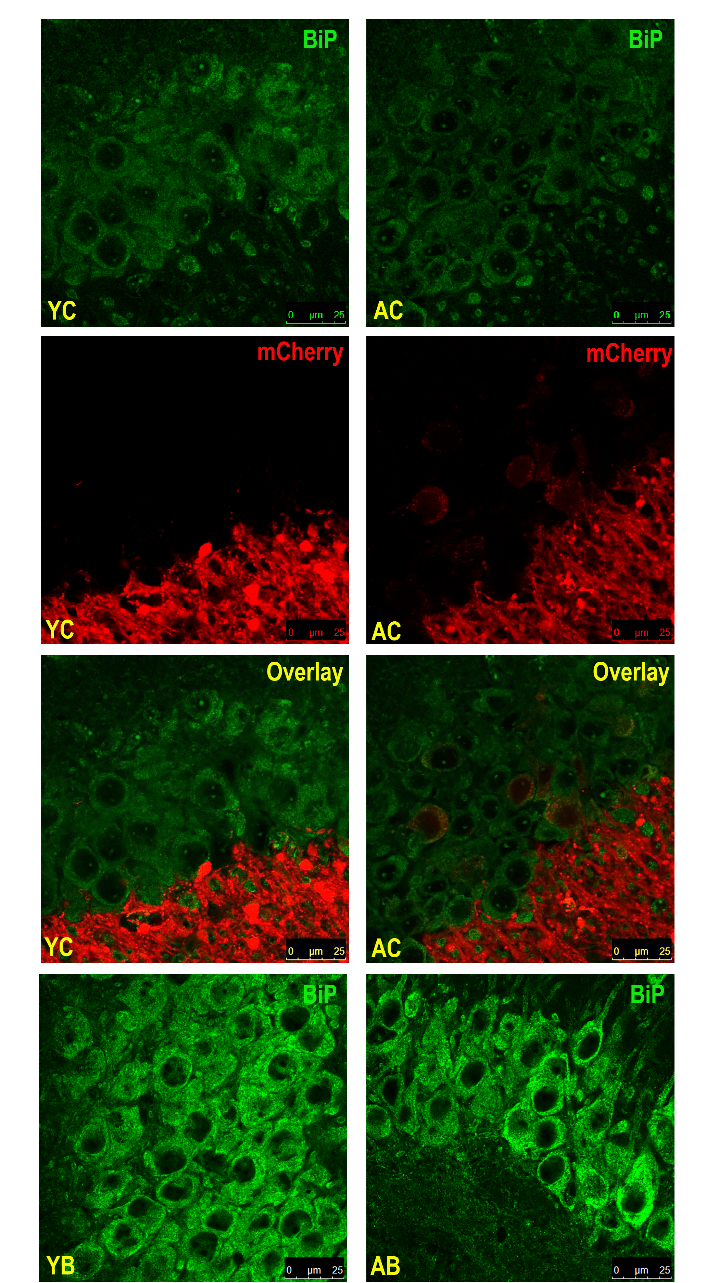


Supplemental Figure 3: Confirmation of BiP-AAV and Control AAV-mCherry targeting in the CA3. A) BiP staining in AAV-BiP young mice B) BiP staining in young AAV-Control mice B’) mCherry staining in young AAV-control mice C) AAV-BiP aged mice D) BiP staining in AAV-BiP aged mice D) BiP staining in aged AAV-Control mice D’) mCherry staining in aged AAV-control mice


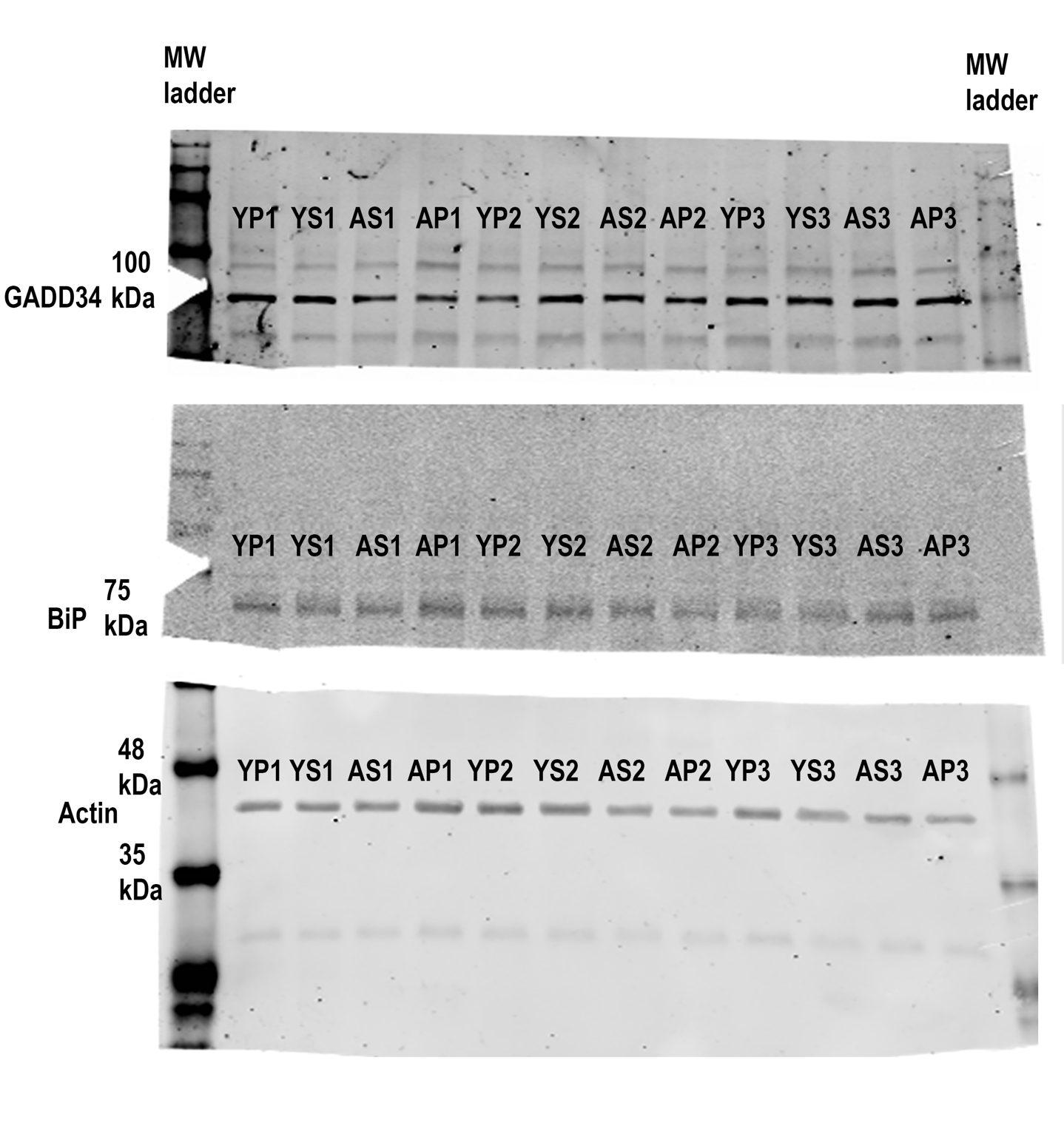


Supplemental Figure 4: Sample Western blot whole membrane image probing for *top*: GADD34

0~100 kDa), *middle*: BiP (~75 kDa), and *bottom*: actin (~42 kDa). Each lane is a different animal, alternating samples from each of the four groups. Membrane was cut roughly along the 60 kDa marker following blocking to utilize two channels for the higher molecular weight proteins using the Odyssey two-channel system. (Abbr: YP = young PBA, YS = young saline, AS = aged saline, AP = aged PBA).

**Supplemental Table 1:** Treatment group by age ANOVA interaction p-values across sleep and wake phenotypes

| **Characteristic** | **Interaction P-value**† | | |
| --- | --- | --- | --- |
|  | **Lights Off** | **Lights On** | **24 Hours** |
| **Sleep/Wake Characteristics** | | | |
| *Wake* |  |  |  |
| Average Bout Duration | 0.081 | **0.041** | **0.047** |
| Bout Number | **0.021** | **0.015** | **0.007** |
| Total Minutes | 0.553 | 0.791 | 0.537 |
| *NREM* |  |  |  |
| Average Bout Duration | **0.039** | **0.012** | **0.008** |
| Bout Number | **0.022** | **0.012** | **0.007** |
| Total Minutes | 0.483 | 0.996 | 0.591 |
| *REM* |  |  |  |
| Average Bout Duration | 0.381 | 0.134 | 0.189 |
| Bout Number | 0.663 | **0.043** | 0.066 |
| Total Minutes | 0.704 | 0.368 | 0.609 |
| †p-value (two-way ANOVA) testing for significant interaction between age-group and treatment; nominally significant interactions (p<0.05) shown in **bold**. | | | |

**Supplemental Table 2: Sleep and wake characteristics over 24 hours of baseline recordings**

| **Characteristic** | **Saline** | | | **PBA** | | | **Aged** | | | **Young** | | |
| --- | --- | --- | --- | --- | --- | --- | --- | --- | --- | --- | --- | --- |
|  | **Young**  **(N=7)** | **Aged**  **(N=9)** | **p**^‡^ | **Young**  **(N=10)** | **Aged**  **(N=9)** | **p**^‡^ | **Saline**  **(N=9)** | **PBA**  **(N=9)** | **p**^‡^ | **Saline**  **(N=7)** | **PBA**  **(N=9)** | **p**^‡^ |
| **WAKE** | | | | | | | | | | | | |
| Bout Duration^§^ | 141.084 ± 22.485 | 94.916 ± 34.896 | 0.1483 | 147.936 ± 37.005 | 160.874 ± 60.342 | 0.9064 | 94.916 ± 34.896 | 160.874 ± 60.342 | **0.0110** | 141.084 ± 22.485 | 147.936 ± 37.005 | 0.9871 |
| Bout Number | 405.286 ± 62.737 | 571.778 ± 160.340 | **0.0144** | 407.400 ± 55.708 | 371.778 ± 90.360 | 0.8716 | 571.778 ± 160.340 | 371.778 ± 90.360 | **0.0013** | 405.286 ± 62.737 | 407.400 ± 55.708 | 1.0000 |
| Total Minutes | 799.048 ± 43.052 | 762.215 ± 62.073 | 0.6894 | 791.640 ± 60.895 | 782.970 ± 86.874 | 0.9917 | 762.215 ± 62.073 | 782.970 ± 86.874 | 0.9092 | 799.048 ± 43.052 | 791.640 ± 60.895 | 0.9958 |
| **NREM** | | | | | | | | | | | | |
| Bout Duration^§^ | 83.215 ± 11.353 | 66.056 ± 15.296 | 0.1065 | 83.757 ± 13.553 | 94.573 ± 16.422 | 0.3765 | 66.056 ± 15.296 | 94.573 ± 16.422 | **0.0012** | 83.215 ± 11.353 | 83.757 ± 13.553 | 0.9998 |
| Bout Number | 407.571 ± 65.248 | 578.111 ± 166.456 | **0.0150** | 415.400 ± 53.029 | 378.778 ± 92.945 | 0.8720 | 578.111 ± 166.456 | 378.778 ± 92.945 | **0.0018** | 407.571 ± 65.248 | 415.400 ± 53.029 | 0.9987 |
| Total Minutes | 572.210 ± 40.921 | 605.933 ± 55.837 | 0.7137 | 583.787 ± 68.854 | 594.215 ± 75.013 | 0.9836 | 605.933 ± 55.837 | 594.215 ± 75.013 | 0.9787 | 572.210 ± 40.921 | 583.787 ± 68.854 | 0.9819 |
| **REM** | | | | | | | | | | | | |
| Bout Duration^§^ | 60.503 ± 10.983 | 50.650 ± 8.640 | 0.4697 | 58.793 ± 17.017 | 61.150 ± 14.096 | 0.9803 | 50.650 ± 8.640 | 61.150 ± 14.096 | 0.3563 | 60.503 ± 10.983 | 58.793 ± 17.017 | 0.9937 |
| Bout Number | 67.143 ± 16.658 | 84.889 ± 16.159 | 0.1602 | 67.400 ± 16.621 | 63.889 ± 16.128 | 0.9658 | 84.889 ± 16.159 | 63.889 ± 16.128 | **0.0494** | 67.143 ± 16.658 | 67.400 ± 16.621 | 1.0000 |
| Total Minutes | 68.743 ± 10.860 | 71.852 ± 8.847 | 0.9740 | 64.573 ± 19.576 | 62.556 ± 14.981 | 0.9903 | 71.852 ± 8.847 | 62.556 ± 14.981 | 0.9787 | 68.743 ± 10.860 | 64.573 ± 19.576 | 0.9371 |
| Two-way ANOVA p-values with Tukey post-hoc correction for multiple comparisons (‡); Average bout duration in seconds (§); Data presented as mean ± SEM, nominally significant differences (p<0.05) shown in **bold**. | | | | | | | | | | | | |

**Supplemental Table 3: Sleep and wake characteristics during baseline lights on (inactive phase) and lights off (inactive)**

| **Characteristic** | **Saline** | | | **PBA** | | | **Aged** | | | **Young** | | |
| --- | --- | --- | --- | --- | --- | --- | --- | --- | --- | --- | --- | --- |
|  | **Young**  **(N=7)** | **Aged**  **(N=9)** | **p**^‡^ | **Young**  **(N=9)** | **Aged**  **(N=9)** | **p**^‡^ | **Saline**  **(N=9)** | **PBA**  **(N=9)** | **p**^‡^ | **Saline**  **(N=7)** | **PBA**  **(N=9)** | **p**^‡^ |
| **Lights On** | | | | | | | | | | | | |
| **Wake** |  | | | | | | | | | | | |
| Bout Duration^§^ | 66.033 ± 16.359 | 54.954 ± 13.322 | 0.6163 | 61.742 ± 11.545 | 76.768 ± 26.848 | 0.2827 | 54.954 ± 13.322 | 76.768 ± 26.848 | 0.0675 | 66.033 ± 16.359 | 61.742 ± 11.545 | 0.9618 |
| Bout Number | 255.857 ± 47.960 | 320.111 ± 70.231 | 0.1012 | 262.700 ± 40.809 | 232.556 ± 50.478 | 0.6149 | 320.111 ± 70.231 | 232.556 ± 50.478 | **0.0080** | 255.857 ± 47.960 | 262.700 ± 40.809 | 0.9937 |
| Total Minutes | 272.762 ± 37.223 | 281.630 ± 34.968 | 0.9670 | 265.200 ± 31.382 | 281.030 ± 47.799 | 0.8039 | 281.630 ± 34.968 | 281.030 ± 47.799 | 1.0000 | 272.762 ± 37.223 | 265.200 ± 31.382 | 0.9777 |
| **NREM** |  | | | | | | | | | | | |
| Bout Duration^§^ | 94.027 ± 17.832 | 54.954 ± 13.322 | 0.1159 | 91.454 ± 15.813 | 102.943 ± 20.208 | 0.4873 | 54.954 ± 13.322 | 102.943 ± 20.208 | **0.0063** | 94.027 ± 17.832 | 91.454 ± 15.813 | 0.9904 |
| Bout Number | 257.714 ± 48.890 | 324.556 ± 71.906 | 0.0931 | 270.400 ± 39.104 | 238.000 ± 53.889 | 0.5763 | 324.556 ± 71.906 | 238.000 ± 53.889 | **0.0107** | 257.714 ± 48.890 | 270.400 ± 39.104 | 0.9649 |
| Total Minutes | 392.771 ± 34.983 | 382.778 ± 27.824 | 0.9507 | 404.580 ± 38.918 | 394.459 ± 44.707 | 0.9342 | 382.778 ± 27.824 | 394.459 ± 44.707 | 0.9097 | 392.771 ± 34.983 | 404.580 ± 38.918 | 0.9175 |
| **REM** |  | | | | | | | | | | | |
| Bout Duration^§^ | 66.072 ± 12.665 | 52.422 ± 4.706 | 0.2343 | 58.832 ± 18.277 | 59.850 ± 15.503 | 0.9986 | 52.422 ± 4.706 | 59.850 ± 15.503 | 0.6764 | 66.072 ± 12.665 | 58.832 ± 18.277 | 0.7217 |
| Bout Number | 51.000 ± 11.818 | 64.444 ± 14.371 | 0.2096 | 52.300 ± 13.573 | 46.556 ± 13.059 | 0.7850 | 64.444 ± 14.371 | 46.556 ± 13.059 | **0.0371** | 51.000 ± 11.818 | 52.300 ± 13.573 | 0.9972 |
| Total Minutes | 54.467 ± 7.466 | 55.593 ± 8.892 | 0.9969 | 50.220 ± 15.031 | 44.511 ± 9.575 | 0.6729 | 55.593 ± 8.892 | 44.511 ± 9.575 | 0.1625 | 54.467 ± 7.466 | 50.220 ± 15.031 | 0.8606 |
| **Lights Off** | | | | | | | | | | | | |
| **Wake** |  | | | | | | | | | | | |
| Bout Duration^§^ | 216.135 ± 37.721 | 134.878 ± 58.885 | 0.1579 | 234.131 ± 73.098 | 244.979 ± 105.376 | 0.9889 | 134.878 ± 58.885 | 244.979 ± 105.376 | **0.0191** | 216.135 ± 37.721 | 234.131 ± 73.098 | 0.9611 |
| Bout Number | 149.429 ± 22.441 | 251.667 ± 110.666 | **0.0186** | 144.700 ± 36.086 | 139.222 ± 46.292 | 0.9977 | 251.667 ± 110.666 | 139.222 ± 46.292 | **0.0046** | 149.429 ± 22.441 | 144.700 ± 36.086 | 0.9988 |
| Total Minutes | 526.286 ± 26.890 | 480.585 ± 40.583 | 0.3156 | 526.440 ± 46.578 | 501.941 ± 75.910 | 0.7336 | 480.585 ± 40.583 | 501.941 ± 75.910 | 0.8178 | 526.286 ± 26.890 | 526.440 ± 46.578 | 1.0000 |
| **NREM** |  | | | | | | | | | | | |
| Bout Duration^§^ | 72.404 ± 7.302 | 58.421 ± 16.328 | 0.3443 | 76.060 ± 16.498 | 86.204 ± 20.686 | 0.5409 | 58.421 ± 16.328 | 86.204 ± 20.686 | **0.0058** | 72.404 ± 7.302 | 76.060 ± 16.498 | 0.9686 |
| Bout Number | 149.857 ± 24.265 | 253.556 ± 112.368 | **0.0190** | 145.000 ± 35.646 | 140.778 ± 47.707 | 0.9990 | 253.556 ± 112.368 | 140.778 ± 47.707 | **0.0052** | 149.857 ± 24.265 | 145.000 ± 35.646 | 0.9988 |
| Total Minutes | 179.438 ± 24.196 | 223.156 ± 38.874 | 0.2864 | 179.207 ± 45.264 | 199.756 ± 67.906 | 0.7864 | 223.156 ± 38.874 | 199.756 ± 67.906 | 0.7289 | 179.438 ± 24.196 | 179.207 ± 45.264 | 1.0000 |
| **REM** |  | | | | | | | | | | | |
| Bout Duration^§^ | 54.935 ± 10.172 | 48.878 ± 13.711 | 0.8771 | 58.754 ± 20.460 | 62.451 ± 16.299 | 0.9584 | 48.878 ± 13.711 | 62.451 ± 16.299 | 0.2970 | 54.935 ± 10.172 | 58.754 ± 20.460 | 0.9625 |
| Bout Number | 16.143 ± 7.010 | 20.444 ± 5.151 | 0.6081 | 15.100 ± 6.350 | 17.333 ± 8.689 | 0.8944 | 20.444 ± 5.151 | 17.333 ± 8.689 | 0.7744 | 16.143 ± 7.010 | 15.100 ± 6.350 | 0.9898 |
| Total Minutes | 14.276 ± 5.027 | 16.259 ± 4.514 | 0.9305 | 14.353 ± 5.859 | 18.044 ± 9.344 | 0.6129 | 16.259 ± 4.514 | 18.044 ± 9.344 | 0.9374 | 14.276 ± 5.027 | 14.353 ± 5.859 | 1.0000 |
| Two-way ANOVA p-values with Tukey post-hoc correction for multiple comparisons (‡); Average bout duration in seconds (§); Data presented as mean ± SEM, nominally significant differences (p<0.05) shown in **bold**. | | | | | | | | | | | | |

**Supplemental Table 4: Baseline delta power data during NREM sleep across treatment within age groups**

| **Characteristic** | **Young** | | | **Aged** | | |
| --- | --- | --- | --- | --- | --- | --- |
|  | **Mean ± SD** | | **p**^†^ | **Mean ± SD** | | **p**^†^ |
|  | **Saline** | **PBA** |  | **Saline** | **PBA** |  |
| **Lights On** | | | | | | |
| **NREM** |  | | | | | |
| 0-1 Hz | 15.756 ± 5.137 | 15.825 ± 9.181 | 1.0000 | 23.948 ± 8.709 | 16.564 ± 5.223 | 0.2651 |
| 1-2 Hz | 79.750 ± 27.058 | 79.695 ± 45.335 | 0.9955 | 123.264 ± 45.588 | 83.033 ± 24.317 | 0.3282 |
| 2-3 Hz | 176.570 ± 59.731 | 166.882 ± 92.497 | 0.9700 | 252.700 ± 72.485 | 179.360 ± 61.933 | 0.5548 |
| 3-4 Hz | 257.874 ± 75.007 | 232.948 ± 137.632 | 0.9744 | 329.041 ± 66.779 | 254.341 ± 93.612 | 0.7174 |
| **Lights Off** | | | | | | |
| **NREM** |  | | | | | |
| 0-1 Hz | 16.538 ± 4.410 | 17.578 ± 8.040 | 0.9954 | 26.071 ± 10.629 | 18.599 ± 5.216 | 0.2707 |
| 1-2 Hz | 82.358 ± 23.460 | 87.638 ± 39.614 | 0.9989 | 134.527 ± 58.171 | 92.211 ± 24.922 | 0.4267 |
| 2-3 Hz | 193.645 ± 51.513 | 200.369 ± 93.808 | 0.9992 | 279.826 ± 99.604 | 207.183 ± 69.980 | 0.7373 |
| 3-4 Hz | 300.177 ± 62.878 | 308.625 ± 166.722 | 0.9980 | 375.297 ± 91.177 | 307.120 ± 114.322 | 0.7959 |
| †pairwise comparisons performed between treatment groups within age; two-way ANOVA with Tukey post-hoc correction for multiple comparisons. | | | | | | |

**Supplemental Table 5: Baseline delta power during NREM sleep across age groups within treatment**

| **Characteristic** | **Saline** | | | **PBA** | | |
| --- | --- | --- | --- | --- | --- | --- |
|  | **Mean ± SD** | | **p**^†^ | **Mean ± SD** | | **p**^†^ |
|  | **Young** | **Aged** |  | **Young** | **Aged** |  |
| **Lights On** | | | | | | |
| **NREM** |  | | | | | |
| 0-1 Hz | 15.756 ± 5.137 | 23.948 ± 8.709 | 0.2069 | 15.825 ± 9.181 | 16.564 ± 5.223 | 0.9980 |
| 1-2 Hz | 79.750 ± 27.058 | 123.264 ± 45.588 | 0.2977 | 79.695 ± 45.335 | 83.033 ± 24.317 | 0.9986 |
| 2-3 Hz | 176.570 ± 59.731 | 252.700 ± 72.485 | 0.5927 | 166.882 ± 92.497 | 179.360 ± 61.933 | 0.9906 |
| 3-4 Hz | 257.874 ± 75.007 | 329.041 ± 66.779 | 0.8146 | 232.948 ± 137.632 | 254.341 ± 93.612 | 0.9806 |
| **Lights Off** | | | | | | |
| **NREM** |  | | | | | |
| 0-1 Hz | 16.538 ± 4.410 | 26.071 ± 10.629 | 0.1294 | 17.578 ± 8.040 | 18.599 ± 5.216 | 0.9970 |
| 1-2 Hz | 82.358 ± 23.460 | 134.527 ± 58.171 | 0.2840 | 87.638 ± 39.614 | 92.211 ± 24.922 | 0.9989 |
| 2-3 Hz | 193.645 ± 51.513 | 279.826 ± 99.604 | 0.6765 | 200.369 ± 93.808 | 207.183 ± 69.980 | 1.0000 |
| 3-4 Hz | 300.177 ± 62.878 | 375.297 ± 91.177 | 0.8432 | 308.625 ± 166.722 | 307.120 ± 114.322 | 0.9928 |
| †pairwise comparisons performed between age groups within treatment, two-way ANOVA with Tukey post-hoc correction for multiple comparisons | | | | | | |

**Supplemental Table 6: Animal Numbers Used for PBA and Saline Experiments**

| Animal Group | Started Treatment | Survived Surgery | Cognitive Testing | Sleep EEG Analyses ≠ | Spectral Analyses |
| --- | --- | --- | --- | --- | --- |
| Young Saline | 11 | 11 | 11 | 7 | 6 |
| Young PBA | 12 | 11 | 11 | 9 | 6 |
| Aged Saline | 11 | 10 | 10 | 9 | 6 |
| Aged PBA | 13 | 11 | 11 | 9 | 6 |
| Animal n’s throughout PBA/Saline treatment experiments; ≠ only mice with good EEG signals were scored for EEG analysis of which the best 6 were analyzed for spectral analyses. | | | | | |
